# The dual specificity phosphatase 2 act as distal regulatory node in T cell signaling

**DOI:** 10.1016/j.isci.2026.116690

**Published:** 2026-07-15

**Authors:** Henrike Bruckmueller, Anangi Balasiddaiah, Sofia Malek, Victoria Tenhaken, Julien Bruckmueller, Jakob Mejlvang, Helene Spangenberg, Farah Syed, Bjarne Johansen, Hanne Kildalsen, Ingolf Cascorbi, Ole-Morten Seternes

**Affiliations:** 1 Institute of Experimental and Clinical Pharmacology, University Hospital Schleswig-Holstein, 24105 Kiel, Germany; 2 Department of Pharmacy, UiT the Arctic University of Norway, 9037 Tromsø, Norway; 3 Solana Research GmbH, 24340 Windeby, Germany

**Keywords:** mitogen-activated protein kinases, MAPK, dual specificity phosphatases, DUSP2, DUSP5, T cells, IL2 production

## Abstract

The mitogen-activated protein kinases (MAPKs) are critical signaling molecules that regulate T cell development, activation, and function. While the MAPK activation is well-characterized, the role of phosphatases as negative regulators remain incompletely understood. This study aimed to comprehensively investigate the role of phosphatases which regulate MAPK pathway activity in human T cells. The dual-specificity phosphatase 2 (DUSP2) was determined as a key negative regulator of ERK1/2, p38, and to a lesser extent of JNK1/2 in human T cells. Loss of DUSP2 is partly compensated by the inducible nuclear phosphatase DUSP5 whose expression is driven by the ERK1/2-dependent transcription factor EGR1, forming a negative feedback loop that modulates MAPK signaling. Functional studies reveal that the ERK1/2-EGR1-DUSP axis regulates the IL-2 production of T cells. Together, these findings suggest a critical role of the ERK1/2-EGR1-DUSP2 axis in fine-tuning MAPK activity and cytokine production during T cell activation.

## Highlights

• DUSP2 is the major negative regulator of MAPK activity in human T cells
• DUSP2 does not function as a STAT3 phosphatase in T cells
• Loss of DUSP2 in T cells is compensated by increased DUSP5 expression
• DUSP2 and DUSP5 act as distal checkpoints controlling IL-2 production in T cells

## Graphical abstract

**Figure:**
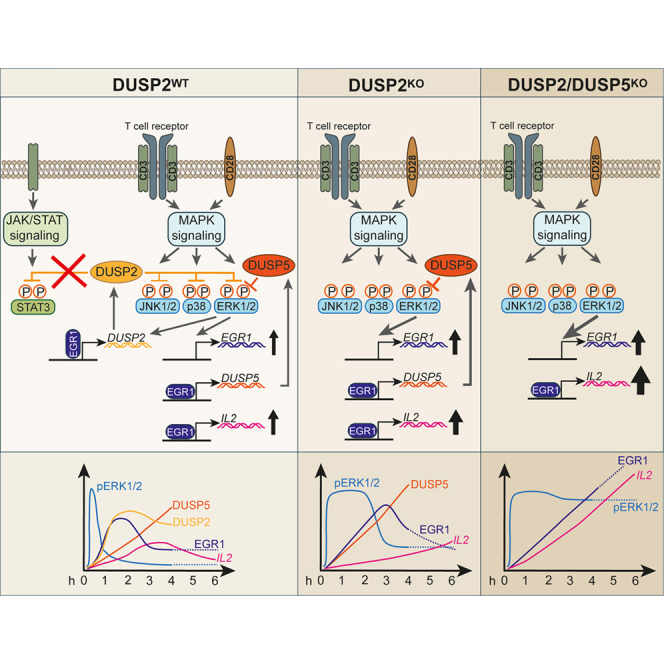

## Teaser

Molecular biology; Immunology; Cell biology

## Introduction

T lymphocytes (T cells) are essential components of the adaptive immune system mediating cell-based immune responses. Aberrant T cell function has been implicated in a wide range of diseases including immunodeficiencies, autoimmune disorders, and cancer. Therefore, a comprehensive knowledge of T cell activation and regulation is required to understand disease processes and potentially develop novel therapeutic approaches.

T cell activation is initiated when the major histocompatibility complex (MHC) on antigen-presenting cells (APCs) presents specific antigens to T cell receptors (TCRs). In addition, T cell costimulatory molecules as CD28 interact with CD80 on APCs to form an immunological synapse. This engagement of the TCR and CD28 leads to activation of intracellular signaling pathways which depend on the rapid phosphorylation and dephosphorylation of multiple signaling and adapter proteins. Among these pathways, the mitogen-activated protein kinase (MAPK) cascade with ERK1/2, p38, and JNK as major components is integral in translating TCR signals into physiological cellular responses such as T cell proliferation and differentiation.^1^ The biological outcome of MAPK activation in T cells is determined by the magnitude and duration of their activation, which is influenced by the nature and strength of the stimuli.^2^ MAPK activation requires phosphorylation of two residues within the conserved threonine (T)-X-tyrosine (Y) motif localized in the kinase activation-loop while dephosphorylation at either residue by protein phosphatases is sufficient to inactivate the kinase. Due to its critical role in TCR signaling the duration of MAPK activation in T cells is tightly controlled by negative feedback loops. This involves direct ERK1/2-mediated phosphorylation of upstream pathway components such as the linker for activation of T cell (LAT) and the induction of phosphatases that target MAPKs themselves.^3^ MAPK phosphatases (MKPs), a subgroup of dual-specificity protein phosphatases (DUSPs), directly inactivate MAPKs by dephosphorylating both residues in the activation loop. This subgroup comprises ten members, which differ in substrate specificity, tissue expression, subcellular localization and catalytic activity with some being constitutively active while others require substrate binding for activation.^4^ Substrate specificity toward the different MAPKs is mediated by specific protein-protein interactions between the kinase-interacting motif (KIM) in the N-terminal non-catalytic domain of MKPs and the common-docking site within the cognate MAPK.^5^

Over the last decades, numerous mechanistic studies and investigations in DUSP-deficient mice have established roles for most DUSPs in immune regulation, with some exhibiting partial functional redundancy.^6^^,^^7^^,^^8^^,^^9^^,^^10^ However, the role of DUSP2, which was initially described as “phosphatase of activated T cells” (PAC-1) remains controversial. Conflicting and unexpected observations have been reported regarding its function in T cell activation and its substrate specificity for different MAPKs.^11^^,^^12^^,^^13^

The aim of the present study was to revisit and further elucidate the role of DUSP2 as a negative regulator of human T cell activation. In this study, functional analysis determined DUSP2 as major negative regulator of MAPK signaling in human T cells whose depletion is compensated for by the induction of the inducible, nuclear phosphatase encoded by *DUSP5* in an ERK1/2-EGR1 dependent manner. Together our data suggest that MAPK signaling during T cell activation, which leads to increased IL2 secretion, is controlled by the negative feedback regulators DUSP2 and DUSP5, both of which themselves are regulated by ERK1/2 induced expression of the transcription factor EGR1.

## Results

### Inducible phosphatases mediate the dephosphorylation of MAP kinases in primary human CD4^+^ T cells

**Figure 1:**
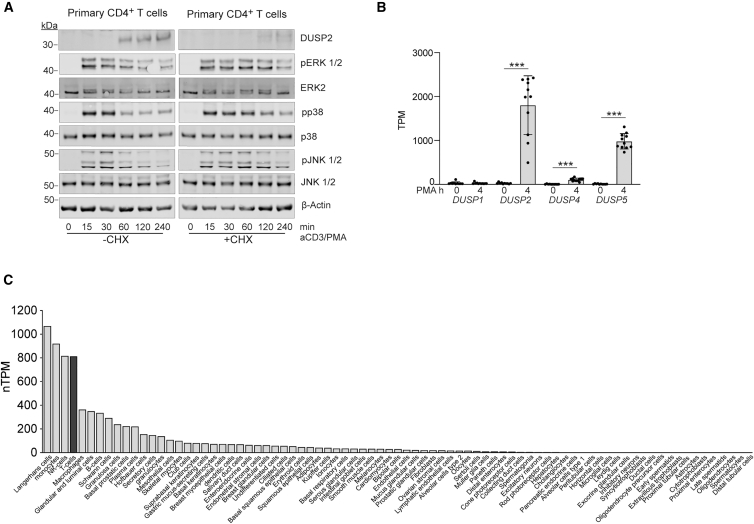
Inducible phosphatases mediate the dephosphorylation of MAPKs in primary human CD4^+^ T cells (A) Primary human CD4^+^T cells were either left untreated or treated with cycloheximide (CHX, 10 μg/mL for 30 min) prior to activation with 5 μg/mL aCD3 and 10 nM PMA. Cell extracts were collected at indicated time points for western blot analysis and probed with the antibodies detecting phosphorylated MAP kinases (*n* = 2). (B) Gene expression data of *DUSP1*, *2*, *4*, *5* from primary human CD4^+^T cells unstimulated (0 h) or stimulated with PMA for 4 h were extracted from the GSE69549^14^ and are presented as mean TPM ±SD from three independent donors (*n* = 10). (C) *DUSP2* mRNA expression data from 81 cell types of 31 datasets from healthy donors, presented as normalized transcripts per million (nTPM). The dataset was derived from the human protein atlas (www.proteinatlas.org). Unpaired student’s *t* test, ∗∗∗*p* < 0.001, PMA = phorbol-12-myristate-13-acetate.

To investigate whether *de novo* protein synthesis is required for dephosphorylation of MAP kinases in activated T cells, primary human CD4^+^ T cells were treated with or without the protein synthesis inhibitor cycloheximide (CHX) 30 min prior to stimulation with aCD3/PMA. Treatment with CHX resulted in sustained phosphorylation of the MAPKs ERK1/2, p38, and JNK1/2 compared to untreated cells, suggesting that the dephosphorylation of all three MAPKs depends on *de novo* protein synthesis (Figure 1A). To determine whether one of the four inducible MAP kinase phosphatases (MKPs) is responsible for MAPK dephosphorylation in naive CD4^+^ T cells, mRNA expression levels of *DUSP1*, *DUSP2*, *DUSP4*, and *DUSP5* of healthy volunteers were extracted from the GSE69549 dataset and compared between unstimulated and activated with PMA (Figure 1B) or aCD3/aCD28 (Figure S1A). While the mRNA expression of *DUSP2*, *4* and *5* was increased after stimulation, the most pronounced elevation in transcript levels was observed for *DUSP2*. This observation was further confirmed at protein level, where an increase in DUSP2 protein in naive CD4^+^ T cells 60 min after stimulation correlated with MAPK dephosphorylation (Figure 1A). Analysis of *DUSP2* mRNA expression across 81 cell types from 31 datasets of healthy donors extracted from the Human Protein Atlas (www.proteinatlas.org) revealed the highest levels in peripheral blood lymphocytes (Figure 1C). Moreover, public available RNAseq data of primary human CD4^+^ and CD8^+^ cells revealed DUSP2 as highest expressed among all detectable DUSPs (Figures S1B and S1C).^14^ Together these findings align with studies that described DUSP2 as phosphatase of activated T cells (PAC-1).^15^ Additionally, the data indicate that inducible phosphatases are essential for dephosphorylation of the classical MAPKs in the T cells.

### DUSP2 dephosphorylates MAPKs and is activated by them *in vitro*

**Figure 2:**
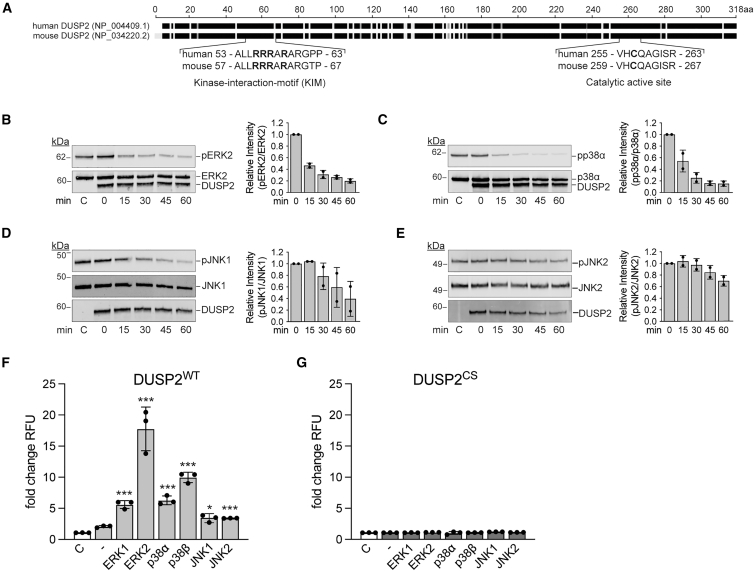
Murine DUSP2 dephosphorylates human MAPKs and is activated by them *in vitro* (A) Protein sequence alignment of human (NP_004409.1) and mouse DUSP2 (NP_034220.2) reveals a pairwise identity of 87.6%, indicated by black bars. Conserved arginine residues within the KIM and the cysteine essential for phosphatase activity are marked in bold. (B–E) Western blot analysis of *in vitro* dephosphorylation assays using 1 μg murine GST-DUSP2 and 1 μg human GST-*p*-ERK2 (B), GST-p-p38 alpha (C), His-*p*-JNK1 (D) or His-*p*-JNK2 (E). The reactions were conducted over 60 min at 37°C, (C) indicates control without addition of DUSP2 protein. Phospho-specific antibodies were used to detect phosphorylated MAPKs, while a-JNK1/2 antibody was used for total JNK1 and JNK2 and an anti-GST antibody for detection of DUSP2, ERK2 and p38α, *n* = 2. Data are presented as mean ± SD of relative signal intensity. (F) Catalytic activity of DUSP2^WT^ was assessed by incubating 1 μg of DUSP2^WT^ protein with 1 μg of the indicated human MAPKs. Phosphatase activity was measured using a DiFMUP phosphatase assay. (G) The same assay as in (F) was performed using the catalytically inactive DUSP2 mutant (DUSP2^CS^). Data are presented as mean ± SD of relative increased fluorescence signal (RFU 60 min/RFU 0 min), *n* = 3, unpaired student’s *t* test, ∗*p* < 0.05, ∗∗∗*p* < 0.001.

While early studies suggested that DUSP2 could dephosphorylate ERK1/2 and p38, findings from murine models and *in vitro* experiments have challenged this observation.^12^^,^^16^^,^^17^ To clarify the substrate specificity of DUSP2, the present study reexamined its activity by incubating recombinant DUSP2 with various phosphorylated MAPKs (ERK2, p38ɑ, JNK1 and JNK2) to assess its dephosphorylation ability. Although murine and human DUSP2 share 87.6% amino acid sequence similarity, we were unable to express the human form in *E. coli*. As a result, these dephosphorylation experiments were conducted using the murine DUSP2 instead (Figure 2A). The results indicated that DUSP2^WT^ could dephosphorylate ERK2 (Figure 2B), p38ɑ, (Figure 2C), and JNK1 (Figure 2D), but not JNK2 (Figure 2E). Since specific interactions between DUSP2 and MAPKs facilitates the catalytic activation of DUSP2, we further tested the ability of various human MAPKs to activate DUSP2.^16^^,^^18^ The findings revealed that ERK1, ERK2, p38ɑ, p38β, JNK1 and JNK2 were all capable to activate DUSP2^WT^ protein, with ERK2 and p38β inducing the highest level of activation (Figure 2F). As expected, none of the tested MAPKs caused dephosphorylation of DiFMUP when incubated with the phosphatase dead mutant of DUSP2 (DUSP2^CS^, Figure 2G). These results indicate that DUSP2 can dephosphorylate a broad range of MAPKs *in vitro*, including ERK 2, p38α, and JNK1.

### DUSP2 dephosphorylates ERK1/2, p38 MAPK, and JNK but not STAT3 in T cells

**Figure 3:**
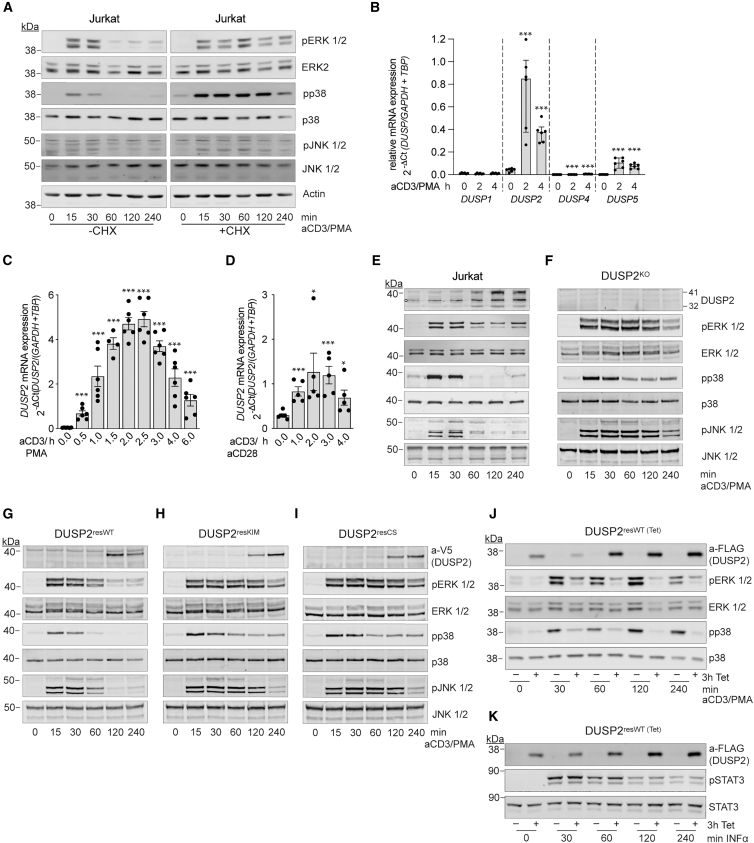
DUSP2 dephoshorylates the MAP kinases ERK1/2, p38 MAPK, and JNK but not STAT3 in T cells (A)Western blot of Jurkat cells either left untreated or pretreated with CHX (10 μg/mL for 30 min) prior to activation with 5 μg/mL aCD3 and 10 nM PMA. Cell extracts were collected at indicated time points and western blots were probed with the antibodies detecting phosphorylated MAP kinases. (B) Relative mRNA expression levels of the inducible DUSPs (DUSP1, 2, 4, 5) in Jurkat cells unstimulated or stimulated for 2 h or 4 h with 250 ng/mL aCD3/and 250 ng/mL PMA (*n* = 3). (C) RT-qPCR analysis was conducted to verify the induction of *DUSP2* mRNA expression in Jurkat^WT^ cells within 6 h after stimulation with 5 μg/mL aCD3 and 10 nM PMA or (D) within 4 h after stimulation with aCD3/aCD28 coated beads. Data are presented as *DUSP2* mRNA expression normalized to geometric mean of *GAPDH* and *TBP* as control (*n* = 3). (E) Western blot analysis of stimulated (5 μg/mL aCD3/10 nM PMA) Jurkat cells showed an inverse correlation of DUSP2 protein levels with MAPK phosphorylation levels after initial induction. (F) DUSP2^KO^ led to sustained MAPK phosphorylation after stimulation. (G) The rescue of Jurkat DUSP2^KO^ cells with wild-type DUSP2 (DUSP2^resWT^) restored the DUSP2 phosphatase function completely. This effect was abrogated in Jurkat cells (H) rescued with DUSP2^resKIM^ or (I) with DUSP2^resCS^ mutants. (J) Stimulation (5 μg/mL aCD3/10 nM PMA) of doxycycline pretreated (2 μg/mL, 3h) DUSP2^resWT (Tet)^ cells showed a DUSP2 dependent dephosphorlyation of ERK1/2 and p38, (K) while STAT3 was not dephosphorylated in a DUSP2 dependent manner in DUSP2^resWT (Tet)^ cells after doxycycline pretreatment (2 μg/mL, 3 h) and INFα (100 ng/mL) stimulation. For all experiments cells were starved overnight, and majority of experiments were performed in at least three replicates. Expression data are presented as mean ± SEM. Unpaired student’s *t* test was calculated compared to unstimulated cells, ∗*p* < 0.05, ∗∗*p* < 0.01, ∗∗∗*p* < 0.001; CHX = cycloheximide, PMA = phorbol-12-myristate-13-acetate; INFα = interferon alpha, TPM = transcripts per million, ° unspecific band.

To further investigate the role of DUSP2 in T cells, we selected the Jurkat T cell line, a well-established model for studying T cell signaling that was also used for the initial characterization of DUSP2.^15^^,^^19^ To confirm that the dynamic of the MAPK pathway regulation in Jurkat cells mirrors that of primary CD4^+^ T cells, we treated Jurkat cells with or without CHX followed by stimulation with aCD3/PMA. In the absence of CHX, MAPK phosphorylation levels initially increased but then steadily declined, whereas CHX treatment resulted in sustained phosphorylation of ERK1/2, p38, and JNK1/2 as observed in primary CD4^+^ T cells (Figure 3A). Furthermore, RT-qPCR results from Jurkat cells revealed an inducible DUSP expression pattern consistent with that observed in primary T cells with most pronounced and likely biologically relevant increase seen for *DUSP2* mRNA which aligns with RNAseq analysis findings in Jurkat cells (Figures 3B and S2A). The significant induction of *DUSP2* mRNA expression was further confirmed in time course experiments of Jurkat cells stimulated either with aCD3/PMA or aCD3/aCD28 coated beads (Figures 3C and 3D). To further investigate DUSP2’s phosphatase function in T cells, we generated Jurkat cell lines in which DUSP2 was knocked-out (DUSP2^KO^) using CRISPR-Cas9 mediated gene editing. Stimulation of Jurkat wild-type and Jurkat DUSP2^KO^ cells revealed DUSP2 as major phosphatase responsible for dephosphorylation of ERK1/2, p38, and JNK1/2 in Jurkat cells (Figures 3E, 3F, S3A–S3G). To confirm that the observed lack of MAPK dephosphorylation in DUSP2^KO^ cells was specifically due to the absence of DUSP2, rescue experiments were performed. To restore the DUSP2 expression at physiological and transient levels, we employed a lentiviral vector in which DUSP2 is driven by the EGR1 promoter. This strategy enabled expression patterns that mimic endogenous DUSP2 regulation by the ERK1/2 signaling pathway. Using this system, we successfully reintroduced both wild-type DUSP2 (DUSP2^resWT^) or mutated versions of DUSP2, either with a disrupted kinase interaction motif (DUSP2^resKIM^) or an active site cysteine replaced with serine (DUSP2^resCS^), at levels comparable to endogenous expression in the DUSP2^KO^ cells. While reintroduction of wild-type DUSP2 fully restored MAPK dephosphorylation (Figure 3G), reintroduction of the DUSP2^resKIM^ or DUSP2^resCS^ mutants failed to restore phosphatase activity against the MAPKs (Figures 3H and 3I).

Beside DUSP2’s role in dephosphorylation of the MAPKs, recent studies have also suggested a potential ability of DUSP2 to dephosphorylate STAT3.^11^ However, stimulation of Jurkat cells with aCD3/PMA, which induces DUSP2 expression, does not lead to increase in STAT3 phosphorylation (Document S2). For further investigations we generated therefore a tetracycline inducible rescue cell line (DUSP2^resWT[Tet]^). In these cells, doxycycline pretreatment followed by aCD3/PMA stimulation induced DUSP2 expression and led to dephosphorylation of ERK1/2 and p38, depending on the presence or absence of DUSP2 protein (Figure 3J). In contrast, stimulation of doxycycline treated DUSP2^resWT(Tet)^ cells with IFN alpha induced STAT3 phosphorylation, which decreased over time. However, STAT3 dephosphorylation was unaffected by the presence or absence of DUSP2 protein (Figure 3K). Taken together, these findings demonstrate that DUSP2 is responsible for the dephosphorylation of ERK1/2, p38, and some extent JNK1/2 in human T cells while STAT3 does not appear to be a direct substrate of DUSP2 in these cells.

### Mutations outside DUSP2s catalytic or N-terminal domain can impair its phosphatase activity

**Figure 4:**
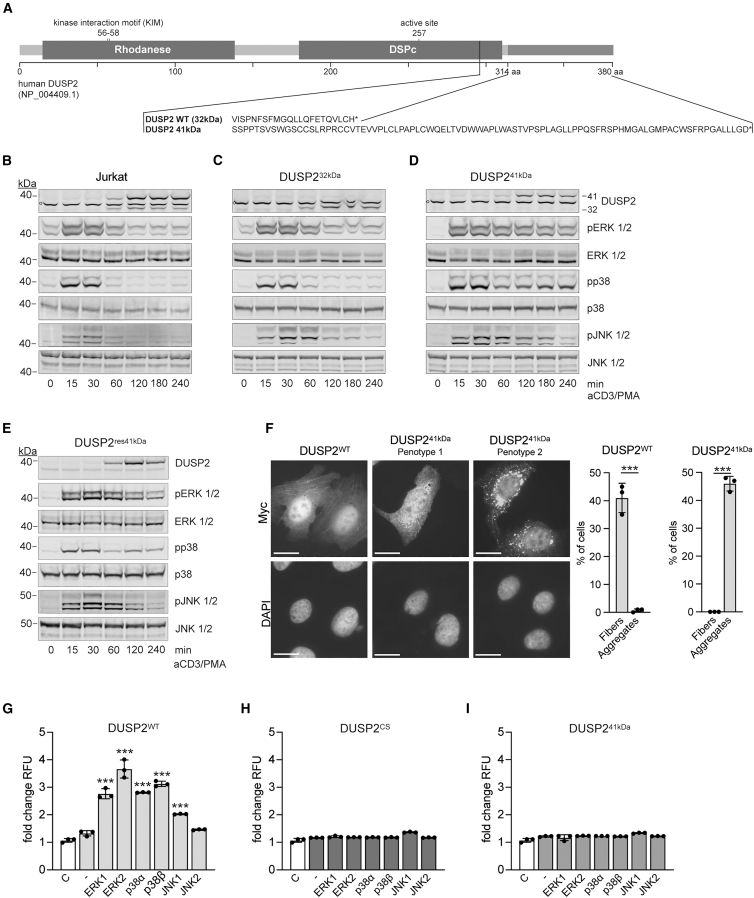
Mutations outside DUSP2 key residues can impair its phosphatase activity (A) Schematic representation of the amino acid sequence of wild-type DUSP2 compared to mutated allele leading to a C-terminal extended DUSP2 protein (41 kDa) of 380aa. (B–D) Western blot analysis of (B) Jurkat cells, (C) single-allele knock-out Jurkat cells expressing DUSP2 32kDA and (D) single-allele knock-out Jurkat cells expressing DUSP2 41kDA after stimulation with 5 μg/mL aCD3/10 nM PMA with the indicated antibodies. (E) Western blot analysis of extracts derived from stimulated (aCD3/PMA) Jurkat DUSP2^KO^ cells rescued with a lentiviral expression vector for the 41 kDa allele of DUSP2 and probed with the indicated antibodies. For all western blot experiments cells were starved overnight. (F) Microscopic analysis showed U2OS cells transfected with myc-tagged expression vectors encoding either DUSP2^WT^ or DUSP2^41kDa^ and stained with an anti-myc antibody to detect DUSP2. The scale bars indicated a length of 25 μm. (G–I) *In vitro* phosphatase assays were performed using 300 ng of (G) wild-type human DUSP2^WT^ protein, (H) catalytically inactive human DUSP2 protein (DUSP2^CS^), or (I) human DUSP2^41kDa^ protein in combination with the indicated MAPKs (1 μg). Data are presented as mean ± SD of relative increased fluorescence signal (RFU 60 min/RFU 0 min), *n* = 3. Unpaired student’s *t* test, ∗∗∗*p* < 0.001. DSP, dual specificity phosphatase; PMA, phorbol-12-myristate-13-acetate.

In addition to the expected 32 kDa DUSP2 band, western blot analysis of Jurkat cells revealed an additional 41 kDa band, previously reported by Ward et al.^15^ (Figure 3E). The absence of both bands in DUSP2^KO^ cells confirmed that both proteins are encoded by the *DUSP2* gene (Figure 3F). A review of the COSMIC database revealed a deletion in one *DUSP2* allele in Jurkat cells (c.877del), resulting in a frameshift at amino acid 293 and the translation of an extended 41 kDa DUSP2 protein (p.V293Sfs∗90, Figure 4A). Given DUSP2’s critical role in MAPK pathway regulation, we sought to determine whether mutations outside its key residues could affect its phosphatase activity. For this purpose, we aimed to compare the ability of the extended 41 kDa DUSP2 protein to dephosphorylate the MAPKs with the dephosphorylation ability of the DUSP2 wild-type (32 kDa) protein. To this end, we generated Jurkat single allele knock-out cells which expressed either exclusively the wild-type DUSP2 allele (DUSP2^32kDa^) or the mutated allele (DUSP2^41kDa^). While stimulation of Jurkat cells (Figure 4B) or of single wild-type allele cells (DUSP2^32kDa^, Figure 4C) led to rapid dephosphorylation of ERK1/2, p38, and JNK1/2, the cells harboring the single mutated allele (DUSP2^41kDa^, Figure 4D) exhibited a sustained MAPK phosphorylation, indicating an impaired phosphatase function of the extended DUSP2 protein. This finding was further validated by rescuing DUSP2^KO^ cells with the extended DUSP2 variant (DUSP2^res41kDa^, Figure 4E). Since the extended DUSP2 variant had an intact catalytic domain of DUSP2, we hypothesized that the C-terminal extension indirectly affects the phosphatase activity, potentially through mis-localization of the mutated protein. To investigate this, we ectopically expressed myc-tagged DUSP2^WT^ and DUSP2^41kDa^ protein in U2-OS cells and subsequently analyzed the subcellular localization of the DUSP2 proteins by immunofluorescence microscopy. While DUSP2^WT^ was diffusely expressed in both cytosol and nucleus, DUSP2^41kDa^ formed aggregates (Figure 4F). Of note, the expression of both DUSP2^KIM^ and DUSP2^CS^ resembled the expression of DUSP2^WT^ (Figure S4A). Cycloheximide chase experiments revealed that DUSP2^41kDa^ appeared to be more stable than DUSP2^WT^ protein (Figure S4B). Furthermore, DiFMUP phosphatase assays showed that DUSP2^41kDa^, similar to DUSP2^CS^, was catalytically inactive in the presence of various MAPKs (Figures 4G–4I). In summary, these results showed that a mutation outside DUSP2s catalytic or N-terminal domain can lead to production of an aberrant protein with impaired phosphatase function potentially due to improper protein folding.

### Loss of DUSP2 in T cells is compensated by DUSP5

**Figure 5:**
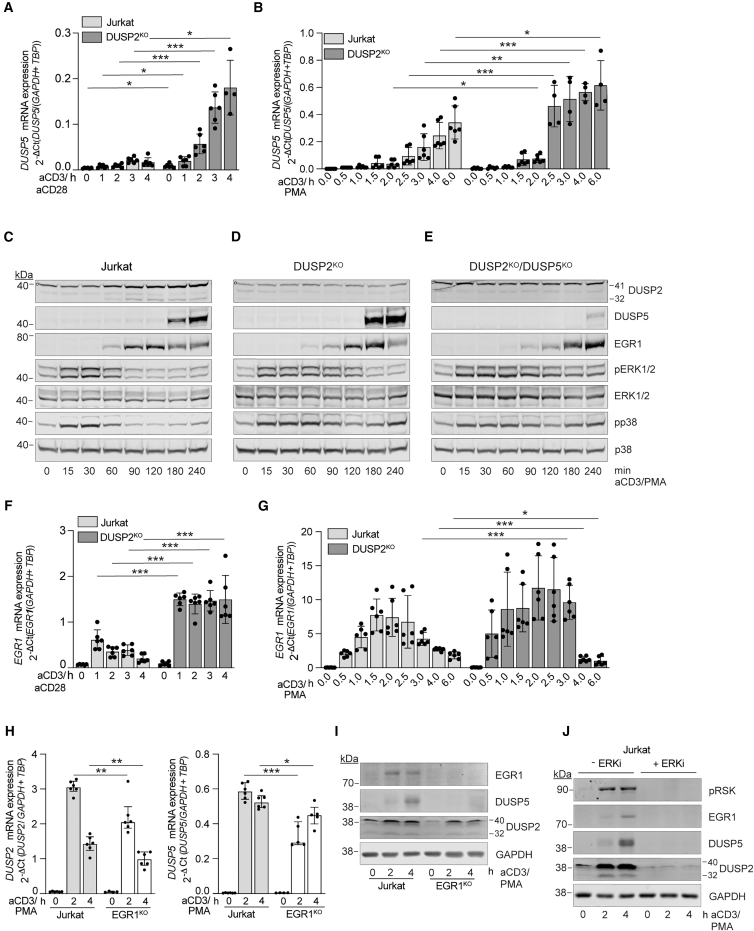
Loss of DUSP2 is compensated by DUSP5 (A) RT-qPCR analysis of *DUSP5* mRNA expression in Jurkat and DUSP2^KO^ cells stimulated with aCD3/aCD28 coated beads (bead/cell ration 1:1) for 0–4 h. (B) RT-qPCR analysis of *DUSP5* mRNA expression in Jurkat and DUSP2^KO^ cells stimulated with 5 μg/mL aCD3/10 nM PMA for 0–6 h. (C–E) Western blots analysis of DUSP2, DUSP5, and EGR1 protein levels and phosphorylation of ERK1/2, and p38 in (C) Jurkat, (D) DUSP2^KO^, (E) and DUSP2/DUSP5^KO^ cells after stimulation with 5 μg/mL aCD3/10 nM PMA. The faint DUSP5 band occurred only in this replicate of the DUSP2/DUSP5^KO^ blot, but was absent in all other replicates (Document S2). (F) RT-qPCR confirmation of *EGR1* mRNA expression in Jurkat and DUSP2^KO^ cells stimulated with aCD3/aCD28 coated beads (bead/cell ration 1:1) for 0–4 h. (G) RT-qPCR confirmation of *EGR1* mRNA expression in Jurkat and DUSP2^KO^ cells stimulated with 5 μg/mL aCD3/10 nM PMA for 0–6 h. (H) Comparison of *DUSP2* and *DUSP5* mRNA expression levels in Jurkat and EGR1^KO^ cells stimulated with 5 μg/mL aCD3/10 nM PMA. (I) Western blot analysis of extracts from Jurkat and EGR1^KO^ cells stimulated with 5 μg/mL aCD3/10 nM PMA and probed with indicated antibodies. (J) Western blot analysis of Jurkat cells either untreated or pretreated with ERK inhibitor (SCH772984, 1 μM, 30 min) prior to stimulation with 5 μg/mL aCD3/10 nM PMA, probed with indicated antibodies. Cells for all experiments were starved overnight prior to stimulation with at least in three replicates. Gene expression data are presented as target gene mRNA expression normalized to geometric mean of GAPDH and TBP as control (*n* = 3). Data are presented as mean ± SD, unpaired student’s *t* test, ∗*p* < 0.05, ∗∗∗*p* < 0.001. PMA = phorbol-12-myristate-13-acetate.

Although DUSP2 knock-out cells exhibited sustained ERK1/2 phosphorylation compared to Jurkat wild-type cells, the phosphorylation levels had decreased 4 h after stimulation (Figure 3F). This observation prompted the question of whether another DUSP could compensate for the loss of DUSP2 function in T cells. The results from RT-qPCR analysis revealed DUSP5 as the only other inducible DUSP considerably induced in stimulated Jurkat cells (Figure 3B). When comparing Jurkat wild-type and DUSP2^KO^ cells, *DUSP5* mRNA expression was significantly higher in DUSP2^KO^ cells upon various T cell stimuli (Figures 5A, 5B, and S2). At protein level, DUSP5 expression increased 3 h after stimulation in both wild-type Jurkat and DUSP2^KO^ cells (Figures 5C and 5D). However, DUSP5 protein levels were higher in DUSP2^KO^ cells than in Jurkat wild-type cells, which correlated with ERK1/2 dephosphorylation but not with dephosphorylation of p38, 3 and 4 h after stimulation (Figures 5C and 5D). To explore DUSP5’s role in the ERK1/2 dephosphorylation at later time points after stimulation, we generated DUSP2/DUSP5 double knock-out cells (DUSP2/DUSP5^KO^). The absence of both DUSPs resulted in sustained ERK1/2 phosphorylation beyond 3 h, while p38 phosphorylation remained unaffected (Figure 5E). Next, we aimed to investigate the mechanism underlying this compensatory regulation. Since previous studies described *DUSP5* as a target of the ERK1/2 downstream transcription factor EGR1, we investigated this mechanism in our Jurkat cell model.^20^ Time-course experiments with various T cell stimuli revealed a significantly higher induction of *EGR1* mRNA expression in DUSP2^KO^ cells with a delayed expression peak compared to wild-type cells (Figures 5F and 5G). A similar trend was observed for EGR1 protein levels in Jurkat DUSP2^KO^ and DUSP2/DUSP5^KO^ cells (Figures 5C–5E). To further investigate the EGR1-dependent regulation of DUSP2 and DUSP5, we generated a Jurkat EGR1^KO^ cell line. RT-qPCR analysis revealed a significant reduction of *DUSP2* and *DUSP5* mRNA levels in EGR1^KO^ cells (Figure 5H). However, at protein level this depletion was only observed for DUSP5 but not for DUSP2 upon EGR1 knock-out (Figure 5I). To further examine whether the expression of DUSP2, DUSP5, and EGR1 was induced by ERK1/2 pathway activation in human T cells, Jurkat cells were stimulated in absence or presence of an ERK1/2 inhibitor. Western blot analysis showed that increased protein levels of DUSP2, DUSP5, and EGR1 during T cell stimulation was completely abolished when ERK1/2 were inhibited. (Figure 5J). Together, these findings suggest that both DUSP2 and DUSP5 control ERK1/2 activity in human T cells, and that loss of DUSP2 is at least partly compensated for by the EGR1-DUSP5 axis.

### DUSP2 and DUSP5 act in concert as intracellular checkpoints regulating IL-2 production in T cells

**Figure 6:**
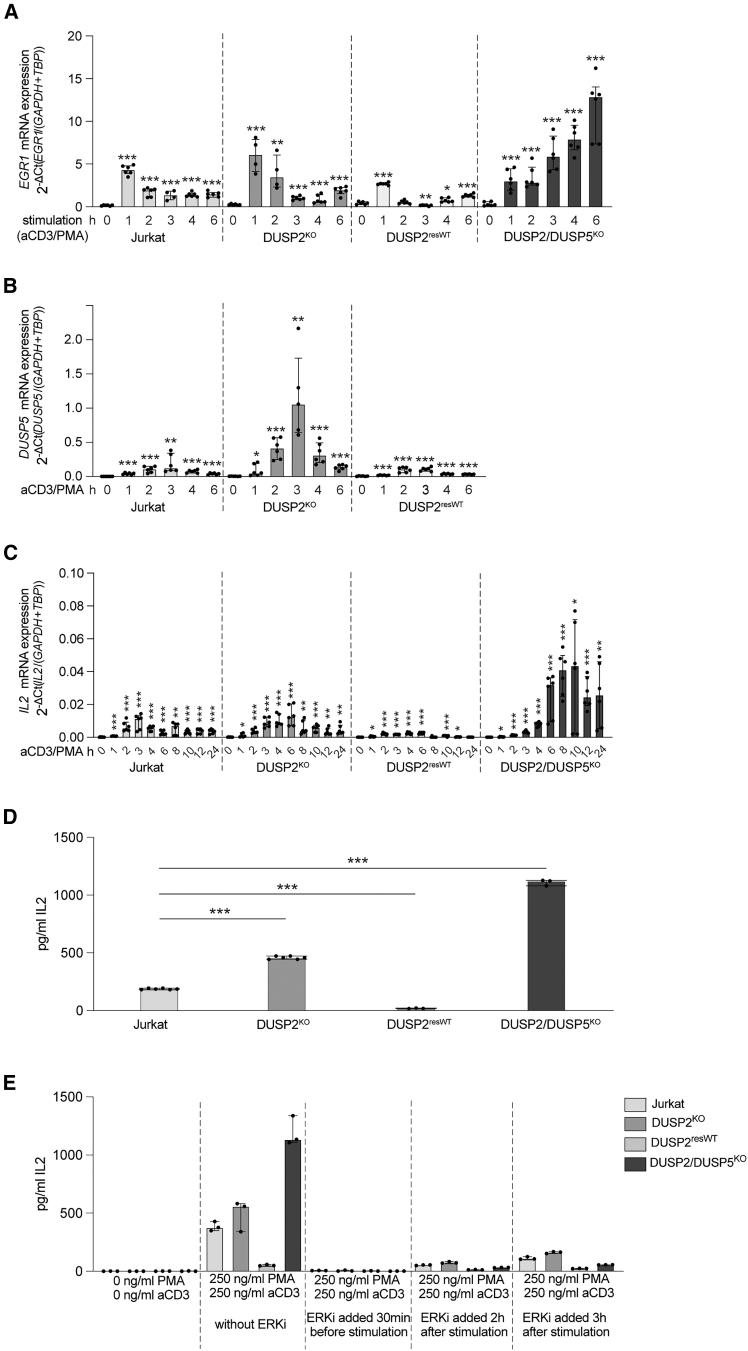
DUSP2 and DUSP5 act in concert to regulate IL2 production in T cells (A–C) RT-qPCR analysis of Jurkat, DUSP2^KO^, DUSP2^resWT^ and DUSP/DUSP5^KO^ cells stimulated with 250 ng/mL aCD3/250 ng/mL PMA. The analysis determined mRNA expression of (A) the *EGR1* (B) *DUSP5* and (C) *IL2*. Gene expression data are presented as target gene mRNA expression normalized to geometric mean of GAPDH and TBP as control and for statistics compared to 0 h of respective cell line. (D) ELISA analysis of IL2 secretion of Jurkat, DUSP2^KO^, DUSP2^resWT^, and DUSP/DUSP5^KO^ cells 24 h after stimulation with 250 ng/mL aCD3/250 ng/mL PMA. (E) ELISA analysis of IL2 secretion of Jurkat, DUSP2^KO^, DUSP2^resWT^, and DUSP/DUSP5^KO^ stimulated for 24 h with 250 ng/mL aCD3/250 ng/mL PMA. Cells were either left unstimulated, stimulated, treated with ERK inhibitor (SCH772984, 1 μM) 30 min before stimulation or treated with ERK inhibitor 2 h or 3 h after stimulation. All experiments were performed at least in three replicates (*n* ≥ 3), data are presented as mean ± SD, unpaired student’s *t* test, ∗*p* < 0.05, ∗∗*p* < 0.01, ∗∗∗*p* < 0.001. PMA = phorbol-12-myristate-13-acetate.

To further investigate the impact of DUSP2 on T cell function and cytokine production, cells with DUSP2 (Jurkat, DUSP2^resWT^) and without DUSP2 activity (DUSP2^KO^, DUSP2/DUSP5^KO^) were stimulated with aCD3/PMA and mRNA expression levels were analyzed over a time course of 6 h (EGR1 and DUSP5) or 24 h (IL2). The mRNA expression levels of *EGR1*, as a downstream transcriptional target of ERK1/2 signaling was higher in DUSP2^KO^ compared to wild-type Jurkat and DUSP2^resWT^ cells (Figure 6A), peaking at 1 h post-stimulation followed by a decline parallel to ERK1/2 activity. In contrast, cells lacking both DUSP2 and DUSP5 (DUSP2/DUSP5^KO^) showed increased *EGR1* mRNA level beyond 2 h post-stimulation indicating prolonged ERK1/2 activity in the absence of both phosphatases. The *DUSP5* mRNA levels were significantly elevated in DUSP2^KO^ cells compared to Jurkat and DUSP2^resWT^ cells (Figure 6B), thereby supporting the suggested compensatory mechanism. Interestingly, *IL2* mRNA expression followed a similar trend to *EGR1*, with low levels in cells with active DUSP2 (Jurkat, DUSP2^resWT^), higher levels in DUSP2^KO^ cells and highest levels in DUSP2/DUSP5^KO^ cells beyond 6 h post-stimulation (Figure 6C). The ELISA analysis confirmed this pattern, showing significantly lower IL2 secretion in cell lines with active DUSP2 (Jurkat, DUSP2^resWT^) compared to those lacking DUSP2 activity and the highest IL2 secretion was observed in DUSP2/DUSP5^KO^ cells (Figures 6D and S5B). This trend was consistent across various stimulation conditions including aCD3/PMA, aCD3/aCD28 and aCD3 alone in different concentrations (Figures S5C–S5E). To further access the role of ERK1/2 in IL2 production, cells were treated with an ERK inhibitor before or after stimulation (aCD3/PMA) (Figure 6E). Pretreatment (30 min) with ERK inhibitor abolished IL2 production in all cells lines thereby indicating the ERK-dependence of IL2 production in Jurkat cells. Furthermore, we observed that *IL2* mRNA expression peaked at 3 h in Jurkat cells, the data showed that full IL2 production required sustained ERK1/2 activity beyond this time point. Data from EGR1^KO^ cells further supported the role of EGR1 as a critical ERK1/2 downstream regulator of IL2 production, as these cells exhibited significantly reduced *IL2* mRNA expression and secretion compared to wild-type Jurkat cells (Figures S5A and S5B). Together, these data indicate that DUSP2 and DUSP5 act as key checkpoints to regulate the duration of ERK1/2 activity and subsequent IL2 production in an EGR1-dependent manner in human T cells.

## Discussion

The MAPK pathway has been extensively studied in the context of T cell signaling but the role of the specific phosphatases responsible for MAPK inactivation remains incompletely understood. Studies performed in murine models led to conflicting results and provided no clear answers in this context.^9^^,^^10^^,^^11^^,^^12^^,^^13^^,^^21^^,^^22^ In the present study, we revisited the role of phosphatases controlling the MAP kinase signaling pathways downstream of the T cell receptor in human T cells.

Our findings demonstrate that *de novo* protein synthesis is required for dephosphorylation of MAPKs following T cell activation, thereby indicating that inducible phosphatases are responsible for this signal termination. Both primary human CD4^+^ cells and Jurkat cells exhibited a moderate upregulation of all four inducible DUSPs, with *DUSP2* showing the most pronounced increase in expression. These observations together with the high *DUSP2* mRNA levels in hematopoietic cells and its earlier description as the phosphatase of activated T cells (PAC-1) support DUSP2’s role as a major negative regulator of MAPK signaling in T cells.^19^

However, the precise function of DUSP2, including its substrate specificity, has been a matter of discussion due to contradictory findings from *in vitro*, *in cellular* and murine studies.^11^^,^^12^^,^^13^^,^^16^ To address these discrepancies, we revisited the regulation of DUSP2 activity and its substrate specificity for MAPKs. Although murine and human DUSP2 share 87.6% amino acid homology we and others were unable to express the full-length human DUSP2 protein in *E*. *coli*.^16^^,^^18^ Using recombinant murine DUSP2 expressed in *E*. *coli*, we observed that all tested MAPKs (ERK1/2, p38ɑ, p38β, and JNK1/2) were able to catalytically activate the phosphatase to varying degrees, with ERK2 and p38β showing the strongest activation. Furthermore, recombinant DUSP2 dephosphorylated ERK2, p38ɑ and JNK1 but not JNK2. These findings are in line with results from earlier *in vitro* studies which identified ERK2 as a physiological substrate of DUSP2 and showed its inability to dephosphorylate JNK2.^16^ In contrast to findings of Zhang et al., our data demonstrated catalytic activation of DUSP2 by both p38 and JNK1.^16^ Moreover, we showed DUSP2’s ability to efficiently dephosphorylate both p38 and JNK1 *in vitro*. The discrepancy in the observed catalytic activation of DUSP2 by p38 and JNK1 may be explained by differences in the substrates used in the phosphatase activity assays in both studies. While Zhang et al. employed the *p*-NPP assay, we used the more sensitive DiFMUP assay in our study which likely enables the detection of weaker activation of DUSP2 by p38 and JNK1.^16^ It is difficult to reconcile why recombinant DUSP2 in our study was able to dephosphorylate p38 *in vitro*, whereas this was not observed by Zhang et al. A key difference between the studies lies in the substrate used: we utilized a GST fusion protein of p38, while Zhang et al. used a His-tagged version. Since GST fusion proteins have a tendency to dimerize, this structural difference may contribute to the observed discrepancy.

To further elucidate DUSP2’s role in human T cells, we were finally able to successfully express recombinant human DUSP2 protein using a wheat germ extract expression system. We could show that recombinant human DUSP2 exhibited catalytic activation by all tested MAPKs, consistent with murine DUSP2 where ERK2 and p38β led to the strongest activation. These results align with overexpression studies demonstrating DUSP2 phosphatase activity against ERK2 and p38.^17^ However, overexpression systems often lead to high, non-physiological protein levels, which might result in off-target effects. To address this limitation, we examined DUSP2’s role and substrate specificity under physiological conditions in primary human CD4^+^ T cells and Jurkat cells.

Our results revealed a robust dephosphorylation of ERK1/2, p38 and to a lesser extent of JNK1/2 upon DUSP2 induction, thereby suggesting that DUSP2 is a crucial negative regulator of MAPK signaling in T cells. This conclusion was further supported by sustained MAPK phosphorylation observed in DUSP2^KO^ cells and cells expressing inactive DUSP2 mutants (DUSP2^resKIM^, DUSP2^resCS^). These findings highlight the role of an intact kinase interaction motif and catalytic center for DUSP2-mediated MAPK dephosphorylation. However, our findings differ from observations in a K/BxN mouse model of inflammatory arthritis where DUSP2 deficiency was associated with increased JNK and reduced ERK and p38 activity.^12^ An explanation for this discrepancy might be that we focused exclusively in our study on the role of DUSP2 in T cells, whereas studies performed in mouse models also analyzed the role of DUSP2 in other immune cell types, such as bone marrow-derived mast cells (BMMCs) and bone marrow-derived macrophages (BMDMs).

A key challenge in studying inducible DUSPs is that they are frequently upregulated by the same signaling pathways they are intended to negatively regulate and individual DUSPs differ in their substrate specificity. Consequently, depletion of one DUSP member can trigger compensatory upregulation of another family member with distinct substrate specificity and/or temporal expression patterns. This complexity is further increased by the fact that different subsets of immune cells display unique patterns of DUSP expression,^11^ and removal of one DUSP in a specific cell type may induce distinct compensatory responses depended on the specific cell type. For instance, in our Jurkat T cell model, depletion of DUSP2 led to increased expression of DUSP5, an ERK1/2-specific phosphatase, resulting in a pronounced yet delayed dephosphorylation of ERK1/2, while allowing sustained phosphorylation of p38 and JNK. In contrast, in mouse models the removal of DUSP2 from BMMCs or BMDMs may instead promote upregulation of another DUSP family members, potentially one targeting ERK1/2 and p38 but not JNK (e.g., DUSP4). As a result, cells lacking DUSP2 in these models may exhibit reduced ERK1/2 and p38 phosphorylation compared to wild-type cells.

The activity of STAT proteins is regulated by phosphorylation. STAT3 is activated through phosphorylation at tyrosine 705, a process mediated by Janus kinases (JAKs), and can be further activated by phosphorylation at serine 727 by MAPKs. While the kinases responsible for STAT3 phosphorylation are well-characterized, the protein phosphatases involved in STAT3 dephosphorylation are less well understood. Most studies suggest that members of the protein tyrosine phosphatases (PTPs) family are primarily responsible for this dephosphorylation.^23^ However, several studies have proposed that members of the DUSP family, including DUSP3,^24^ DUSP4,^25^ DUSP21,^26^ and DUSP2,^11^ may also negatively regulate STAT3 phosphorylation. Among these, only DUSP3, DUSP21, and DUSP2 have been shown to directly dephosphorylate STAT3. DUSP4, on the other hand, appears to inhibit STAT3 phosphorylation indirectly by blocking the activation of JAK.^25^ In contrast to previous studies, our data do not support STAT3 as a physiological substrate of DUSP2 in human T cells.^11^ STAT3 phosphorylation was unaffected by DUSP2 expression even in interferon alpha stimulated cells. The studies suggesting that DUSPs can dephosphorylate STAT3 directly have been conducted in HEK293 cells, where the protein is expressed transiently at very high levels. It is well-known that overexpression can obscure the true substrate specificity of the phosphatase. In contrast, our study utilized DUSP2 expression levels closer to endogenous levels, which may better reflect the physiological context and may provide a more accurate representation of substrate specificity.

In the first study describing DUSP2 in Jurkat cells, Ward et al. detected two bands on the western blot, one at the expected size of 32 kDa, and another protein of 41 kDa using a DUSP2 antibody.^15^ Similarly, in our Jurkat E6.1 cells we observed this expression pattern and identified by DNA sequence analysis that Jurkat cells carry one wild-type and one mutated DUSP2 allele. This mutation results in a frameshift at amino acid 293 thereby translating into an extended 41 kDa DUSP2 protein compared to the wild-type DUSP2 protein of 32 kDa. Using single allele knock-out cell lines and the DUSP2^res41kDa^ cell line we demonstrated that the extended 41 kDa DUSP2 protein exhibits impaired phosphatase activity. This was further confirmed in *in vitro* assays where the DUSP2^41kDa^ protein behaved like the catalytically inactive DUSP2^CS^ mutant.

Given the localization of the mutation, we hypothesized that the impaired phosphatase function might result from altered intracellular protein localization and found that the mutated protein formed cytosolic aggregates with higher protein stability compared to the DUSP2^WT^ protein. These results indicate that mutations lying outside of either the N-terminal kinase interaction motif or the catalytic domain of DUSP2 can lead to production of an aberrant protein with impaired phosphatase function potentially due to improper protein folding. This observation is of particular interest, as recent studies identified DUSP2 to be frequently mutated in hematological neoplasms such as diffuse large B cell lymphoma with no distinct mutational hotspot in the functional domains.^27^^,^^28^^,^^29^ Given that DUSP2 regulates all three canonical MAP kinases and that mutations outwith the kinase interaction motif or catalytic domain can impair DUSP2’s function, cancer-specific DUSP2 mutations might affect MAPK signaling in cancer cells. This might potentially influence both cancer progression and cancer cells’ response to therapy which should be further investigated in future studies.

Our results also revealed a compensatory mechanism involving DUSP5 which together with DUSP2 regulates the MAPK dynamics and IL2 production in activated T cells in an EGR1-dependent manner. While DUSP2 serves as the primary negative regulator of p38 and JNK kinases, both DUSP2 and DUSP5 jointly modulate ERK1/2 activity through temporally distinct feedback mechanisms. The differential induction kinetics of these phosphatases, DUSP2 peaking at 60 min versus DUSP5 appearing 2–3 h post-stimulation, creates a sequential braking system that shapes duration of ERK1/2 activity in T cells. The ERK1/2-EGR1 axis emerges as a central regulatory hub for this regulation which is supported by earlier studies describing EGR1 as a critical regulator for DUSP2 and DUSP5 transcription.^13^^,^^20^^,^^30^

Although both phosphatases require EGR1 for transcription, the DUSP2 protein abundance seemed to be unaffected by EGR1 depletion. This suggests a potential post-transcriptional regulation of DUSP2 as recently described for DUSP6.^31^ For DUSP6, it was shown that its protein translation is under stringent and selective control of specific eukaryotic initiation factors which are themselves controlled by upstream MAP kinase. Such a decoupling of transcriptional and translational regulation may also enable rapid regulation of DUSP2 protein abundance in response to upstream signaling in T cells. Our findings linking the sustained ERK1/2 activity beyond 2-3 h to IL2 production support the “two-tier” model proposed by Chang and coworkers which suggests that ERK signaling in the late phase of T cell activation is essential for IL2 production.^32^ While several transcription factors are known for their role in IL2 induction such as the nuclear factor of activated T cells (NFATs) family members, octamer-binding protein (OCT-1) or forkhead box P3 (FOXP3), only EGR1 and the activator protein 1 (AP-1) are directly controlled by ERK1/2 (31). Our data suggest that EGR1 not only controls the expression of MAPKs negative regulators but also contributes to IL2 expression during late-phase of T cell activation. This indicates that EGR1 may function as part of the ERK1/2 signaling pathway in the late phase of T cell activation and thereby supporting the proposed “two-tier” model. Our data may also suggest that DUSP2 and DUSP5 could function as distal regulatory nodes essential for full T cell activation. A respective function is supported by our observation that depleting expression of both DUSP2 and DUSP5 enables IL2 production even in absence of a co-stimulatory signal (Figure S5E).

The Jurkat T cell model used in this study, originally selected for its high IL2 production upon stimulation, provided valuable insights into MAPK regulation.^33^^,^^34^ Our finding of a single functional DUSP2 allele in Jurkat cells may explain their elevated IL2 production, as reintroducing wild-type DUSP2 under control of the EGR1 promoter resulted in reduced IL2 levels. Furthermore, one could hypothesize that Jurkat cells might be addicted to a single functional DUSP2 allele since we were unable to generate cells with two functional alleles of DUSP2 using gene-editing.

In conclusion, this study highlights DUSP2 as central negative regulator of MAPK signaling in human T cells with its loss being compensated by DUSP5 in an ERK1/2- and EGR1-dependent manner. Together, DUSP2 and DUSP5 form a coordinated negative feedback loop that fine-tunes MAPK signaling during T cell activation, ultimately influencing IL2 production. Given DUSP2’s restricted expression in hematopoietic cells and the role of DUSP2 and DUSP5 in T cell activation, these phosphatases might represent interesting immunomodulatory targets for potential pharmacological interventions aiming to modulate T cell activity. Future studies should explore the therapeutic potential of targeting DUSP2 and DUSP5 in immune-related diseases and cancer.

### Limitations of the study

The main limitation of this study is that most experiments were conducted using the Jurkat T cell model system with only a limited number of experiments performed in primary human CD4^+^ T cells and none in murine models. Moreover, a potential influence of sex, age, ancestry, or ethnicity of cell lines or primary cell donors could not be completely excluded. However, the tightly regulated expression of DUSPs (MKPs) through negative feedback loops in response to MAPK activation makes the Jurkat cell model advantageous for dissecting the roles of various individual phosphatases. Such detailed analysis would be much more challenging in murine models due to the complexity of *in vivo* systems. Although Jurkat T cells are a leukemia-derived cell line with numerous mutations, we demonstrated that the MAPK pathway and DUSP regulation upon stimulation in Jurkat cells are comparable to those observed in primary human CD4^+^ T cells. Therefore, we think that the Jurkat cell model provides a reliable system to study the negative regulation of MAPK signaling and IL2 production in distal T cell signaling.

## Resource availability

### Lead contact

Requests for further information and resources should be directed to and will be fulfilled by the lead contact, Ole-Morten Seternes (ole-morten.seternes@uit.no).

### Materials availability

All raw data reported in this study will be shared by the lead contact upon request. Newly generated materials associated with this study will be made available on request, but we may require a payment and/or a completed materials transfer agreement if there is potential for commercial application.

### Data and code availability

• RNA-seq data have been deposited as BioProject:PRJNA1391914 and are publicly available as of the date of publication. Source data and replicates of all figures containing western blot data have been deposited at Mendeley as Document S2 at Mendeley Data: https://doi.org/10.17632/wdzbkc55br.1. This study analyzes also existing, publicly available data, accessible as NCBI Gene Expression Omnibus (GEO): GSE69549.^14^
• This study does not report original code.
• Any additional information required to reanalyze the data reported in this study is available from the lead contact upon request.

## Acknowledgments

We thank Irina Naujoks, Kerstin Viertmann, Anna Jürgensen, and Britta Schwarten for their excellent technical assistance. We thank Ingvild Mikkola for her thoughtful input and for the critical discussions of the research findings, Axel Scheidig for his helpful suggestions regarding the wheat germ based protein expression method, and Stephen M. Keyse for critical reading of the manuscript. This study was supported by the 10.13039/100008730Norwegian Cancer Society (grant 198119-2019) and the Norwegian Children Cancer Foundation (project 190033 and 250007). Victoria Tenhaken was supported by a grant of the Medical Faculty of the University of Kiel.

## Author contributions

Conceptualization, H.B., A.B., and O.-M.S.; formal analysis, H.B., A.B., and J.B.; funding acquisition, H.B., I.C., and O.-M.S.; investigation, H.B., A.B., S.M, V.T., J.B., J.M., H.S., F.S., B.J., H.K., and O.-M.S.; methodology, H.B., S.M, J.B., B.J., and O.-M.S.; resources, H.B., I.C., and O.-M.S.; supervision, H.B. and O.-M.S.; visualization, H.B.; writing – original draft, H.B., A.B., and O.-M.S.; writing – review and editing, H.B., S.M, V.T., J.B., J.M., H.S., F.S., B.J., H.K., I.C., and O.-M.S.

## Declaration of interests

The authors declare no competing interests.

## STAR★Methods

### Key resources table

**Table:** 

REAGENT or RESOURCE	SOURCE	IDENTIFIER
**Antibodies**		

a-Pan-Actin (rabbit pAb)	CST	#4968; RRID:AB_2313904, 1:1000
a-β-Actin (mouse mAb) (AC-15)	Santa Cruz	sc-698790; RRID:AB_1119529
a-CD3	Tonbo Biosciences	40-0038-U500 RRID:AB_2621439
a-CD28	Enzo Life Sciences	70-0289-U500
a-*c*-myc-tag (rabbit pAb)	Sigma-Aldrich	SAB4301136, 1:1000
a-DUSP2 (rabbit pAb)	Sigma-Aldrich	hpa071920, 1:400
a-DUSP2 (sheep pAb)	Custom made	1:400
a-DUSP2 (mouse mAb) (4O21)	Santa Cruz	sc-32776; RRID: 2094883, 1:1000
a-DUSP5 (rabbit mAb)	Abcam	Ab200708, 1:1000
a-EGR1 (rabbit mAb) (44D5)	CST	#4154, 1:1000; RRID:AB_2097035
a-pERK1/2 (rabbit mAb) (Thr202/Tyr204)(D13.14.4E)	CST	#4370, 1:1000; RRID:AB_2315112
a-pERK1/2 (rabbit pAb) (Thr202/Tyr204)	CST	#9101, 1:1000; RRID:AB_331646
a-ERK1/2 (mouse mAb) clone #216703	R&D Systems	MAB1576; RRID:AB_2140121, 1:1000
a-ERK1/2 (mouse mAb) (C-9)	Santa Cruz	sc-514302; RRID:AB_2571739, 1:1000
a-FLAG (mouse mAb) (M2)	Sigma-Aldrich	F1804; RRID:AB_262044, 1:1000
a-GAPDH (mouse mAb) (0411)	Santa Cruz	sc-47724; RRID:AB_627678, 1:7000
a-GST (rabbit pAb) (Z-5)	Santa Cruz	sc-459; RRID:AB_63158, 1:1000
a-HSP90 (rabbit mAb) (C45G5)	CST	#4877, 1:1000; RRID:AB_2233307
a-pJNK1/2 (rabbit pAb) (Thr183, Tyr185)	Promega	V793A, 1:1000
a-pJNK1/2 (rabbit mAb) (Thr183/Tyr185), (81E11)	CST	#4668, 1:1000; RRID:AB_823588
a-pJNK1/2 (rabbit pAb) (Thr183/Tyr185)	CST	#9251, 1:1000; RRID:AB_331659
a-JNK1/2 (mouse mAb) (D-2)	Santa Cruz	sc-73455864, 1:1000
a-JNK1/2 (rabbit pAb)	CST	#9252, 1:1000; RRID:AB_2250373
a-pp38 (rabbit pAb) (Thr180/Tyr182)	CST	#9211, 1:1000; RRID:AB_331641
a-p38 (rabbit pAb)	CST	#9212, 1:1000; RRID:AB_33071
a-p38 (mouse mAb) (A-12)	Santa Cruz	sc-7972, 1:1000
a-p38 clone 27	BD Transduction Labs	612169, 1:1000
a-p90RSK (rabbit mAb) (Thr359) (D1E9)	CST	#8753, 1:1000; RRID:AB_2783561
a-RSK (rabbit pAb) (C-21)	Santa Cruz	sc-231, 1:200; RRID:AB_632367
a-pSTAT3 (rabbit mAb) (Y785)(D3A7)XP	CST	#9145S, 1:1000; RRID:AB_2491009
a-STAT3 (mouse mAb) (124H6)	CST	#9139S, 1:1000; RRID:AB_331757
a-V5-tag (rabbit pAb)	Sigma-Aldrich	SAB1306079, 1:1000
Goat anti-Rabbit IgG (H + L) Cross-Adsorbed Secondary Antibody, Alexa Fluor™ 488	ThermoFisher Scientific	A-11008
F(ab')2-Goat anti-Rabbit IgG (H + L) Cross-Adsorbed Secondary Antibody, Alexa Fluor™ 488	ThermoFisher Scientific	A-11070
IRDye® 800CW Donkey anti-Rabbit IgG Secondary Antibody	LI-COR Biosciences	926-32213, 1:20000
IRDye® 800CW Goat anti-Rabbit IgG Secondary Antibody	LI-COR Biosciences	926-32211, 1:10000
IRDye® 800CW Goat anti-Mouse IgG Secondary Antibody	LI-COR Biosciences	926-32210, 1:10000
IRDye® 680RD Goat anti-Rabbit IgG Secondary Antibody	LI-COR Biosciences	926-68071, 1:10000
IRDye® 680RD Goat anti-Mouse IgG Secondary Antibody	LI-COR Biosciences	926-68070, 1:10000

**Bacterial strains**		

E.coli (BL21)	ThermoFisher Scientific	
E. coli DH5-α-T1	ThermoFisher Scientific	
E. coli Stbl-3	ThermoFisher Scientific	

**Biological samples**		

Human: primary CD4^+^ T cells	Blood bank, UNN, Tromsø, Norway	

**Chemicals, peptides and recombinant proteins**		

DMEM	Gibco, ThermoFisher Scientific	41965–039
RPMI-1640	Gibco, ThermoFisher Scientific	21875–034
Fetal bovine serum (FBS)	Bio&Sell	FBS.S0615
X-VIVO-15	Lonza	02-060Q
Lymphoprep	Stem cell technology	#07811
Glucose	Carl Roth	HN06.2
HEPES	Sigma-Aldrich	H3375
Human IL2 premium grade	Myltenyi Biotec	130-097-744
Dynabeads Human T-Activator CD3/CD28	Gibco, ThermoFisher Scientific	11131D
Human IFN-α 2b Recombinant Protein	CST	#36000
PMA (phorbol 12-myristate-13-acetete)	Enzo Life Sciences	BML-PE160-0005
Ampicillin	Sigma-Aldrich	
Cycloheximide (CHX)	Sigma Aldrich	C4859-1 ML
Doxycycline hydrochlorid	MedChemExpress	HY-N0565A
Kanamycin	Sigma Aldrich	K1876
Puromycin	Sigma Aldrich	P8833
SCH772984	MedChemExpress,	HY-50846
BamH1-HF	New England Biolabs	R3136S
EcoRI	New England Biolabs	R0101S
Kpn1-HF	New England Biolabs	R3142S
XhoI	New England Biolabs	R0146S
XbaI	New England Biolabs	R0145S
GST-ERK1	MRCPPU Reagents	DU1509
His-ERK2	Novus Biologicals	NBP1-30309
GST-*p*-ERK2	MRCPPU Reagents	DU650
GST-p38 alpha	MRCPPU Reagents	DU979
GST-p-p38 alpha	MRCPPU Reagents	DU979
GST-p38 beta	MRCPPU Reagents	DU1791
His-JNK1	MRCPPU Reagents	DU700
His-*p*-JNK1	MRCPPU Reagents	DU700
His-JNK2	Novus Biologicals	NBP1-98883
His-*p*-JNK2	MRCPPU Reagents	DU699
Glutathione Superflow Agarose	ThermoFisher Scientific	25236
Glutathione	ThermoFisher Scientific	78259
MagicMark™ XP Western-Proteinstandard	Invitrogen, ThermoFisher Scientific	LC5602
SeeBlue Plus2 Pre-Satined Standard	Invitrogen, ThermoFisher Scientific	LC5925
Chameleon® Duo Pre-stained Protein Ladder	LI-COR Biosciences	928–60000
NuPAGE LDS Sample buffer	ThermoFisher Scientific	NP0008
NuPAGE Sample Reducing agent	ThermoFisher Scientific	NP0009
Nitrocellulose membrane	LI-COR Biosciences	926–31092
Intercept (PBS) Blocking Buffer	LI-COR Biosciences	927–70001
DifMUP	Invitrogen, ThermoFisher Scientific	D6567
Beta glycerolphosphate	Carl Roth	
Brij-35	Sigma-Aldrich	Brij L23 solution
DAPI	Sigma-Aldrich	MBD0020
DTT (Dithiothreitol)	biomol GmbH	04010.5
EGTA (ethylene glycol-bis(beta-aminoethyl ether) – N,N,N′,N′- tetraacetic acid)	Merck	108435
EDTA (Ethylenadiaminetetraacetic acid)	Merck	108421
Imidazole	Carl Roth	3899.1
MgCl_2_	Carl Roth	KK36.1
NaCl	Carl Roth	HN00.2
NaF	Carl Roth	2618.1
Polybrene	Santa Cruz	sc-134220
Protease inhibitors cocktail	Roche	04693132001
Sodium orthovanadate	Sigma-Aldrich	S6508-10G
Sucrose	Carl Roth	9097.1
Tetra-sodium pyrophosphate	Carl Roth	0269.1
Tris	Carl Roth	A411.3
Tris/HCl	Carl Roth	9090.4
Triton X-100	Sigma-Aldrich	93443
Vectrashield mounting medium	Vector Laboratories	H-1000

**Critical commercial assays**		

CD4^+^ T cell isolation Kit	Myltenyi Biotec	130-096-533
Venor GeM OneStep Kit	Minerva Biolabs	11–8025
Cell Line Nucleofector Kit V	Lonza	VCA-1003
TransIT-293 transfection reagent	Mirus Bio	MIR2704
Gateway cloning kit	ThermoFisher Scientific	12535–019
In Fusion HD Cloning Kit	Takara Bio Europe SAS	102518
Nucleo Bond Xtra Midi Kit	Macherey-Nagel	740410.50
Protein Research Kit G16	CFS	CFS-PRK-G16
E.Z.N.A. Total RNA Kit I	Omega Bio-tek	R6834-02
Quick RNA miniprep kit	Zymo Research	R1055
High-Capacity cDNA Reverse Transcription Kit	ThermoFisher Scientific	4368813
TaqMan Universal Mastermix II, with UNG	ThermoFisher Scientific	4440039
ROTI Nanoquant	Carl Roth	K880
Pierce^TM^ BCA Protein assay	ThermoFisher Scientific	23225

**Deposited data**		

RNAseq data	Quinn et al.^14^ 2015	NCBI Gene Expression Omnibus (GEO): GSE69549
RNAseq data	This study	BioProject: PRJNA1391914
Document S2	This study	Mendely Data: https://doi.org/10.17632/wdzbkc55br.1

**Experimental models: Cell Lines**		

Human: Jurkat cells (clone E6.1)	ATCC	TIB-152
Human: HEK 293T cells	Dharmacon	DHARHCL4517
Human: U-2OS cells	ATCC	HTB-96

**Oligonucleotides**		

Primers used in this study, see Table S1		

**Recombinant DNA**		

pGEX-mDUSP2	Perander et al.^18^ 2017	
pGEX-mDUSP2-CS	Perander et al.^18^ 2017	
pSG5-DUSP2-WT-myc	Perander et al.^18^ 2017	
pSG5-DUSP2-KIM-myc	Perander et al.^18^ 2017	
pSG5-DUSP2-CS-myc	Perander et al.^18^ 2017	
pSG5-DUSP2-41 kDa-myc	This study	
pENTR1A	ThermoFisher Scientific	A10462
pENTR1A-V5	This study	
pENTR1A-DUSP2-WT-V5	This study	
pENTR1A-DUSP2-KIM-V5	This study	
pENTR1A-DUSP2-CS-V5	This study	
pENTR1A-DUSP2-41 kDa-V5	This study	
pAd5EGR1	Kidger et al.^35^ 2017	
pLenti-CMV-Puro-DEST	gift from Eric Campeau & Paul Kaufman (Addgene)^36^ 2009	#17452
pLenti-EGR1-Puro-DEST	This study	
pLenti-EGR1-DUSP2-WT-Puro-DEST	This study	
pLenti-EGR1-DUSP2-KIM-Puro-DEST	This study	
pLenti-EGR1-DUSP2-CS-Puro-DEST	This study	
pLenti-EGR1-DUSP2-41 kDa-Puro-DEST	This study	
pLix402	gift from David Root (Addgene)	#41394
pENTR1A	Thermo Fischer Scientific	A10462
pENTR1A-V5	This study	
pENTR-FLAG	This study	
pLix402-(tet)-DUSP2-WT-FLAG	This study	
psPAX2	gift from Didier Trono (Addgene)	12260
pMD2.G	gift from Didier Trono (Addgene)	12259
pEU-E01-DHFR	CFS	
pEU-E01-GFP	CFS	
pEU-E01-GST-PS-MCS-N3	CFS	
pEU-E01-GST-PS-DUSP2-WT	This study	
pEU-E01-GST-PS-DUSP2-CS	This study	
pEU-E01-GST-PS-DUSP2-41 kDa	This study	
pZero-Blunt	ThermoFisher Scientific	K270020
pSpCas9(BB)-2A-EGFP (px458)	gift from Feng Zhang (Addgene)^37^ 2013	48138
px458–1.1	This study	
px458-1.1-DUSP2	This study	
px458-1.1-DUSP5	This study	
px458-1.1-EGR1	This study	

**Software and algorithms**		

GraphPad Prism 10.2	GraphPad, Bosten, USA	
Image Studio Lite 5.2.5	LI-COR Biosciences	
Empiria Studio Version 1.3.0.83	LI-COR Biosciences	
FastQC v0.11.8	www.bioinformatics.babraham.ac.uk/projects/fastqc	
Trimmomatic, version 0.39	Bolger et al.^38^ 2014	
TopHat (v2.1.0)	Love et al.^39^ 2014	
DESeq2 (v3.10)	Love et al.^39^ 2014	

**Other**		

BD FACSAria III cell sorter	BD Biosciences	
Bioanalyzer	Agilent Technologies	
Cover glasses (12 mm)	Paul Marienfeld GmbH	0117520
DeltaVision Elite microscope	GE Healthcare Life Sciences	
Electroporation cuvette (4 mm)	VWR	732–1137
Gene Pulser Xcell electroporation system	Bio-Rad Laboratories	
Infinite M200 Pro microplate reader	Tecan	
LS columns	Myltenyi Biotec	130-042-401
Nucleofector 2b device	Lonza	
QuadroMACS separator	Myltenyi Biotec	
QuantStudio^TM^ 7 Flex Real-Time PCR Instrument	ThermoFisher Scientific	
Odyssey CLx reader	LI-COR Biosciences	
SigmaPrep spin column	Sigma-Aldrich	sc1000
Spark	Tecan	

### Experimental model and study participant details

#### Ethical statement

All procedures performed in studies involving human material were in accordance with the ethical standards of the institution. Leukocytes of two anonymized healthy donors were isolated from buffy coats obtained from the Blood bank at the UNN, in Tromsø, Norway. The anonymized healthy donors gave written, general consent for the use of their blood in research. Since the donors were anonymized no informations an their sex, age, ancestry or ethnicity were available which is a limitation of this study. The potential influence of these factors can therefore not be compeletly excluded.

#### Cell culture

Jurkat cells (clone E6.1, ATCC, TIB-152), originally derived from a 14-year-old boy with acute lymphoblastic leukemia, were cultivated in RPMI-1640 medium (Gibco) supplemented with 10% heat inactivated fetal bovine serum (FBS, Bio&Sell), 25 mM glucose and 10 mM HEPES (Sigma-Aldrich), while HEK 293T cells (Dharmacon, DHARCL4517), originally derived from a kidney of a human femal embryo, and U-2OS cells (ATCC, HTB-96), originally derived from a moderately differentiated sarcoma of a 15-year-old, white, female osteosarcoma patient, were cultured in DMEM (Gibco) supplemented with 10% FBS. Cells were routinely monitored for mycoplasma contamination using the Venor GeM OneStep Kit (Minerva Biolabs). The Jurkat and Hek 293T cell lines have been authenticated by the Institut of medical biology, UIT The Arctic University of Norway, Tromsø, Norway) using the ANSI/ATCC ASN-0002-2011 standardized short tandem repeat (STR) profiling for authentication of human cell lines. Primary CD4^+^ T cells were isolated from buffycoats of healthy donors which were provided by the Universitetssykehuset Nord-Norge HF (UNN, Tromsø, Norway) with permission. Peripheral blood mononucleor cells (PBMCs) were isolated by density gradient centrifugation using Lymphoprep (Stem cell technology). Thereafter, CD4^+^ T cell were isolated using the CD4^+^ T cell isolation kit from Myltenyi Biotec with LS columns and the QuadroMACS separator according to the manufacturer’s protocol. After isolation, the primary CD4^+^ T cells were cultured in X-VIVO-15 medium (Lonza) supplemented with 5% human serum (home made) and 10U/mL IL2 (Milteny Biotec) for normal culture and 30U/mL IL2 (Milteny Biotec) for expansion. Expansion of primary CD4^+^ T cells was performed with Dynabeads Human T-Activator CD3/CD28 for T cell expansion and activation (ThermoFisher) according to manufacturer’s protocol. All cells were cultured at 37°C at 5% CO2.

### Method details

#### Plasmids and constructs

The pGEX-mDUSP2 and pGEX-mDUSP2-CS vectors encoding for mouse full-length or CS mutated DUSP2 as well as the Myc-tagged human DUSP2 wild-type (WT), KIM, and CS in pSG5 vector were generated as described earlier.^18^ The pENTR1A-V5 and pENTR1A-3xFLAG vectors was generated by ligation of two annealed oligoes encoding the V5-tag into the pENTR1A vector (A10462, ThermoFisher Scientific) linearized with the restriction enzymes XhoI and XbaI (New England Biolabs GmbH). pENTR1A-DUSP2-WT-V5, pENTR1A-DUSP2-KIM-V5 and pENTR1A-DUSP2-CS-V5 were generated by subcloning of the EcoRI/XhoI fragments encoding the various DUSP2 variants into EcoRI/XhoI sites of pENTR1A-V5 ensuring that the C-terminus of DUSP2 was in frame with the V5-tag. The DUSP2-41 kDa variant was synthesized by ThermoFisher as Gene-Art and cloned as a EcoR1/XhoI fragment into the pENTR1A-V5 lead to pENTR1A-DUSP2-41 kDa-V5. The pSG5-DUSP2-41 kDa-myc was generated by subcloning, therefore the EcoRI/XhoI DUSP2-41 kDa fragment from the pENTR1A-DUSP2-41 kDa-V5 was ligated into the pSG5-myc vector. The vector pLenti-EGR1-Puro-DEST was generated by exchanging the CMV promoter in pLenti-CMV-Puro-DEST (17452, Addgene) with the EGR1 promoter from the pAd5EGR1 vector of Kidger et al. 2017^35^ using the In-Fusion cloning system (Takara Bio Europe). The lentiviral expression vectors for the various DUSP2 variants (DUSP2^resWT^, DUSP2^resKIM,^ DUSP2^resCS^, DUSP2^res41kDa^) were generated by gateway recombination (12535–019, ThermoFisher) using the variant specific entry vectors (pENTR1A-DUSP2-V5) to generate the variant specific destination vectors (pLenti-EGR1-DUSP2-Puro-DEST). For tetracyclin inducible expression of DUSP2^resWT (Tet)^ cDNA containing a C-terminal FLAG tag was cloned in a pENTR vector and then recombined into the pLix402 vector (41394, Addgene) using the gateway LR reaction. The vectors for the wheat germ cell-free protein expression system were generated by subcloning of the various DUSP2 variant fragments (DUSP2-WT, DUSP2-CS, DUSP2-41 kDa) from the pSG5-DUSP2-myc vectors into the BamH1/Kpn1 linearized pEU-E01-GST-PS-MCS-N3 vector using the In-fusion HD cloning Kit (Takara) with the following primers: DUSP2-InF_F and DUSP2-InF_R for DUSP2-WT and DUSP2-CS and DUSP2-InF_F and DUSP2_41 kDa_InF_R for DUSP2-41 kDa (see Table S1).

#### Generation of knock out cell lines by CRISPR/Cas9 genomic editing

To generate gRNA constructs, the pSpCas9(BB)-2A-EGFP (px458) vector was purchased from Addgene (48138)^37^ and mutated in SPpCas9 (K848A/K1003A/R1060A) as described by Slaymaker et al. 2016 to generate the px458–1.1 vector with the improve specificity and reduce off-target activity.^40^^,^^41^ The specific gRNAs were designed using the CHOPCHOP web tool (https://chopchop.cbu.uib.no)^42^ and the following guides for DUSP2: 5′-GCTGCTGCACGAGACCCGCG-3′, DUSP5: 5`-GAGCGAGCCGCGCACGTTCG-3′ and EGR1: 5′-GCGGCCAGTATAGGTGATGG-3` were cloned into the px458–1.1 vector according to the protocol from Ran et ai.^37^ To generate Jurkat E6.1 knock out cell lines, the cells were electroporated with the px458–1.1 expression vectors containing the respective gRNAs using the Cell Line Nucleofector Kit V and a Nucleofector 2b device with the X-01 program according to manufacturer’s protocol (Lonza). Forthy-8 h after electroporation the EGFP-expressing cells were sorted using a BD FACSAria III cell sorter (BD Biosciences) and distributed into 96-well plates as single cells. Cells with either single or double allele DUSP2^KO^, double allele DUSP5^KO^, double allele EGR1^KO^ or double allele DUSP2/DUSP5^KO^ were identified by immunoblotting. Thereafter, genomic DNA was isolated from the knock-out cells and the region of interest was amplified by PCR. The PCR products were cloned into the pZero-Blunt vector (ThermoFisher Scientific) and indels were verified using Sanger sequencing.

#### Generation of stable rescue cell lines

Lentiviral transduction was also used to stably reintroduce DUSP2 wild-type or variants into the CRISPR/Cas9 DUSP2 knock out (DUSP2^KO^) Jurkat T cells. In order to generate the lentiviral particles, HEK293T cells were transfected with the pLenti-EGR1-Puro-DEST or pLix402 expression vectors, containing the coding sequences of the various DUSP2 variants along with packaging plasmid psPAX2 (12260, Addgene) and pMD2.G (12259, Addgene) using Transit-LT1 transfection reagents according to manufacturer’s protocol. Supernatant was harvest 48 and 72 h post transfection. Supernatants containing the viral particles were steril-filtered, mixed with polybrene (10 μg/mL) and added to the Jurkat T DUSP2^KO^ cells. After 48 h the transduced cell lines (DUSP2^resWT^, DUSP2^resKIM,^ DUSP2^resCS^, DUSP2^res41kDa^, DUSP2^resWT[Tet]^) were selected with puromycin (1 μg/mL).

#### Cell activation experiments

To analyze the expression of DUSPs and EGR1 as well as their effects on protein phosphorylation and mRNA expression primary CD4^+^ T cells or Jurkat cell lines were starved overnight (RPMI-1640 medium with 0.2% h. i. FBS, 25 mM glucose, 10 mM HEPES) and then seeded in 6 well plates (5∗10^6^ cells per time point for protein analysis) and (2∗10^6^ cells per time point for mRNA analysis). Cells were stimulated for different durations either with 5 μg/mL soluble anti-CD3 (aCD3, Tonbo Biosciences) plus 10 nM phorbol 12-myristate-13-acetete (PMA) or 1 μg/mL soluble aCD3 plus 1 μg/mL PMA or 250 ng/mL soluble anti-CD3 plus 250 ng/mL PMA or 1 μg/mL soluble aCD3 plus 1 μg/mL soluble anti-CD28 (aCD28, Tonbo Biosciences) or with Dynabeads Human T-Activator CD3/CD28 (Gibco) in a cell to beads 1:1 ratio depending on the experiment. The respective stimulations and stimulation durations are indicated in the Fig. legends. For the cycloheximide (CHX) experiments primary CD4^+^ T or Jurkat wild-type cells were pretreated with or without a final concentration of 10 μg/mL CHX for 30 min before adding the respective stimulation. For ERK inhibitor experiments primary CD4^+^ T or Jurkat wild-type cells were pretreated with or without a final concentration of 1 μM ERK1/2 inhibitor SCH772984 (MedChemExpress) for 30 min before adding the respective stimulation. For experiments in the tetracycline inducible DUSP2 recue wild-type cell line (DUSP2^resWT [Tet]^) cells were pretreated with 2 μg/mL doxycycline (MedChemExpress) for 3 h followed either by stimulation with 5 μg/mL aCD3 plus 10 nM PMA or by 100 ng/mL Interferon alpha (INFɑ) for the indicated time points. Cells were harvested and pelleted at the indicated time points, washed once with 1xPBS, snap frozen in liquid nitrogen and stored at −80°C until further use for western blot of mRNA expression analysis.

#### Protein stability experiments

To compare the protein stability of the human DUSP2 wild type protein with various DUSP2 variants (DUSP2^KIM^, DUSP2^CtS^ and DUSP2^41kDa^) 5∗10^5^ Hek293T cells were seeded per well in a 6 well plate and were transfected after 24 h with 1 μg of the pSG5-DUSP2-myc vectors containing the various DUSP2 variants (DUSP2^WT^, DUSP2^KIM,^ DUSP2^CS^, DUSP2^41kDa^) using the *Trans*IT-293 Transfection Reagent according to the manufacturers protocol. Untransfected cells were included as controls. Twenty-four hours after transfection cells were treated with 10 μM cycloheximide to inhibit protein synthesis. The CHX-treated cells were harvested at the indicated time points (0 h, 1 h, 2 h, 3 h, 4 h, 5 h, 6 h) and washed once with 1xPBS, snap frozen in liquid nitrogen and stored at −80°C until further use for western blot analysis.

#### Western Blot analysis

For western blot analysis cell pellets were lysed in MKK lysis buffer (20 mM Tris/HCl (pH 7.0), 1 mM EGTA, 1 mM EDTA, 1% (w/v) Triton X-100, 1 mM sodium orthovanadate, 50 mM sodium fluoride, 5 mM tetra-sodium pyrophosphate, 0.27 M sucrose, 10 mM beta glycerolphosphate) supplemented with 1x protease inhibitors cocktail (Roche). After pelleting cellular debris (13000 rpm, 10 min, 4 °C) the supernatant was collected and the total protein amount was determined using the Pierce™ BCA Protein assay according to the manufacturer protocol. Samples containing 20 μg of total protein were used for all blots, except for DUSP2 detection, for which 100 μg of total protein was loaded. The samples were mixed with 4x NuPAGE LDS Sample buffer and 10x NuPAGE Sample Reducing agent before heating to 70°C for 10 min. Sample were loaded on 12%–15% SDS-polyacrylamide gels and transferred to nitrocellulose membrane (LI-COR) using a wet blot system. The membrane was blocked for 1 h with Intercept (PBS) Blocking Buffer (LI-COR) at room temperature (RT) before incubating with the primary antibody overnight at 4°C. Next day, the membrane was washed three times with TBST (TBS plus 0.1% v/v Tween 20), incubated with the corresponding secondary antibody for 1 h, washed again three times with TBST and scanned with the AutoScan function of an Odyssey CLx reader (LI-COR) analyzed using Image Studio Lite 5.2.5 and Empiria software 1.3.0.83 (LI-COR). The used antibodies are listed in key resources table and in the legends of the source data files.

#### Subcellular localization

To determine the subcellular localization of human DUSP2 variants 3∗10^6^ U2OS cells were transfected with 20 μg of pSG5-DUSP2-WT-myc, pSG5-DUSP2-KIM-myc, pSG5-DUSP2-CtS-myc or pSG5-DUSP2-41 kDa-myc vector by resuspending cells with 1x PBS supplemented vector DNA. Cells were then transferred to 4 mm electroporation cuvette (732–1137; VWR) and subjected to a single pulse of 250 V and 250 μFd using the Gene Pulser Xcell electroporation system (Bio-Rad Laboratories). Electroporated cells were seeded on 12 mm cover glasses in DMEM +10% FBS and were grown for 24 h. For immunofluorescent staining, cells were washed in 1x PBS, fixed in methanol for 20 min at −20°C and after rinsing, incubated with primary anti-myc-tag antibodies (SAB4301136; Sigma Aldrich) for 1 h at RT, followed by rinsing, and incubation with Alexa 488–conjugated goat anti-rabbit (A-11070, ThermoFisher) for 1 h at RT. Next, cells were stained with DAPI and finally mounted in vectrashield mounting medium (Vector Laboratories). Cells were examined and photographed using a DeltaVision Elite microscope (GE Healthcare Life Sciences).

#### Expression of recombinant DUSP2 protein

The recombinant GST-tagged mouse DUSP2^WT^ and DUSP2^CS^ protein were expressed and purified as described in Perander et al. 2017 using the pGEX-mDUSP2 and the pGEX-mDUSP2-CS vectors.^7^ The recombinant GST-tagged human DUSP2^WT^, DUSP2^CS^ and DUSP2^41kDA^ proteins were expressed in a cell free system using the Protein Research Kit G16 (CFS-PRK-G16, Cell Free Sciences) according to manufacturer’s protocol. In brief, for MIDI-scale protein expression (1.2 mL total translation volumn) 5 μL of the respective pEU-E01-GST-PS-DUSP2 plasmid (1 μg/μL) was mixed with 0.63 μL SP6 polymerase (80 U/μL), 0.63 μL RNase inhibitor (80 U/μL), 5 μL NTP mix (25 mM), 10 μL 5× Transcription Buffer LM and 28.8 μL nuclease free water and incubated at 37 °C for 6 h. After completion, the RNA quality was accessed on a 1.5% agarose gel followed by the translation reaction. For translation, 46 μL of the transcription reaction was gently mixed with 50 μL wheat germ extract (WEPRO7240) and 0.2 μL creatine kinase (20 mg/mL). To ensure a high efficacy of the translation reaction the bilayer surface was increased by dividing the translation mixture and the 1xSUB-AMIX SGC translation buffer in four flat bottom vials. Twenty-four μL of translation mixture was transferred to the bottom of each vial prefilled with 275 μL 1xSUB-AMIX SGC translation buffer. Each vial was close with a lid to prevent evaporation and incubated for 20 h at 20 °C before gently mixing by pipetting.

#### Protein purification

Purification of GST-tagged protein was performed using Glutathione Superflow Agarose (25236, ThermoFisher Scientific). After washing the Glutathione Superflow Agarose twice with equilibration buffer, pH 8.5 (20 mM Tris, pH 7.5, 2 mM EDTA, 2 mM EGTA, 1% Triton X-100, 0.03% Brij-35), it was restored to a 50% suspension. Hundred μl of the suspension were mixed with 550 μL translation mixture, pH was adjusted to 8.5–9.0 with 50 mMTris-HCl pH 9.0 and the mix was incubated on a rotating wheel overnight at 4°C. After equilibration, the sample was loaded on a SigmaPrep spin column (SigmaAldrich), washed six times with 400 μL washing buffer pH 8.5 (20 mM Tris, pH 7.5, 2 mM EDTA, 2 mM EGTA, 0.03% Brij-35) and finally the GST-tagged protein was eluted using 100 μL elution buffer pH 8.5 (wash buffer pH 8.5 with 20 mM glutathione). The flowthrough fraction was collected, and the elution procedure was repeated three more times. The protein amounts were determined using Bradford reagent (ROTI Nanoquant, Carl Roth) according to the manufacturers protocol.

#### *In vitro* dephosphorylation assay

For the *in vitro* dephosphorylation assay 0.8 μg of the phosphorylated human MAP kinases *p*-ERK2 (DU650), p-p38 alpha (DU979), *p*-JNK1 (DU700) or *p*-JNK2 (DU699) were added to a reaction tube containing 80 μL of phosphatase reaction buffer (100 mM Tris, 100 mM NaCl, 5 nM MgCl_2_, 10 mM DTT, pH 7,5) and were incubated at 37°C. After taking the control sample, 1 μg of mouse DUSP2^WT^ protein was added to the reaction mix and additional samples (12 μL each) were taken after 0 min, 15 min, 30 min, 45 min and 60 min. All samples were directly stored on ice in tubes containing 4x NuPAGE LDS Sample buffer and 10x NuPAGE Sample Reducing agent. The phosphorylation states and amounts of MAPKs were assessed by western blot using the following antibodies: a-GST (Santa Cruz, sc-459), a-pERK1/2 (CST, #4370), a-pp38 (CST, #9211) a-pJNK1/2 (CST, #9251), a-JNK1/2 (Santa Cruz sc-7345).

#### *In vitro* phosphatase assays

To determine the potential differences of MAP kinases to activate mouse and human DUSP2 phosphatase activity *in vitro* phosphatase assays were performed in absence and presence of human MAP kinases. The fluorogenic substrate, 6,8-difluoro-4-methylumbelliferyl phosphate (DiFMUP, Invitrogen) was used in a final concentration of 10 μM in reaction buffer containing 50 mM imidazole, 10 mM DTT, pH 7.5. The reactions were carried out in 96 well black wall microtiter plate in 200 μL reaction buffer and either 1 μg mouse or 300 ng human DUSP2 protein with or without 1 μg ERK1 (DU1509), ERK2 (NBP1-30309), p38 alpha (DU979), p38 beta (DU1791), JNK1 (DU700) or JNK2 (NBP1-98883). Each sample was measured in triplicates and the fluorescence of the product was detected on a Infinite M200 Pro microplate reader (Tecan) with setting of 358/5 nm for extinction and 450/5 nm for emission 25°C every 2 min for 60 min. Control sample was the corresponding reaction buffer.

#### RNA isolation, reverse transcription, quantitative real-time PCR

Total RNA was extracted from Jurkat cell pellets using the E.Z.N.A. Total RNA Kit I (R6834-02; Omega Bio-tek, Norcross, USA) and 400 ng total RNA were reverse transcribed by the High-Capacity cDNA Reverse Transcription Kit (#4368813, ThermoFisher Scientific) according to the manufacturer’s protocol. MRNA expression levels of *DUSP2*, *DUSP5*, *EGR1* and *IL2* were determined on a QuantStudio™ 7 Flex Real-Time PCR Instrument (ThermoFisher Scientific) using the relative gene expression protocol with TaqMan Universal Mastermix II, with UNG (#4440039, ThermoFisher Scientific) and *GAPDH* and *TBP* as endogenous controls. Gene expression levels were analyzed using the ^ΔΔ^Ct method with *GAPDH* and *TBP* as endogenous controls.^43^ The target genes expression levels were normalized to the geometric mean of the expression of the reference genes GAPDH and TBP. For each condition at least three biological replicates were analyzed. TaqMan assay IDs are given in Table S1.

#### RNAseq

For RNASeq, per sample 2∗10^6^ Jurkat wild-type or DUSP2^KO^ cells were starved overnight and either left unstimulated (0h) or stimulated 2 h with aCD3/aCD28 coated beads (ThermoFisher) in a bead/cell ratio 1:1. After cell harvesting RNA was extracted from whole using the Quick RNA miniprep kit (Zymo Research) according to the manufacturers protocol and RNA quality was determined using Bioanalyzer (Agilent Technologies, Inc. CA, USA). Library preparation with TrueSeq RNA library preparation (Illumina, San Diego, USA) and RNA-seq, paired end, 2 × 150bp with 30 million reads per sample using the NovoSeq 6000 Sequencing system (Illumina, San Diego, USA) were performed by Macrogen Inc. (Seoul, South Korea). Read quality was checked with FastQC v0.11.8 (www.bioinformatics.babraham.ac.uk/projects/fastqc) and trimming was performed using Trimmomatic, version 0.39.^38^ Individual sequences were aligned to the human GR.Ch38.97 (Homo sapiens GR.Ch38.97.gtf) reference genome using TopHat (v2.1.0) and transcripts were counted and normalized using DESeq2 (v3.10) from the Bioconductor package.^39^ The Wald test was used for determining differentially regulated genes between Jurkat cells 2h vs. 0h; DUSP2^KO^ 2h vs. 0h, DUSP2^KO^ 0h vs. Jurkat cells 0h and DUSP2^KO^ 2h vs. Jurkat cells 2h followed by *p*-value correction using the Benjamini-Hochberg procedure and calculation of log2 fold changes. Corrected *p*-values below 0.05 were considered as statistically significant.

#### IL2 ELISA experiment

For determination of IL2 secretion 2∗10^6^ Jurkat cells were seeded in 1 mL per well of 6 wells plates in growth medium and stimulated with 250 ng/mL aCD3 plus 250 ng/mL PMA for 24 h. Supernatants were collected and IL2 levels were determined using the Qunatikine ELISA human IL2 immunoassay from R&D systems according to the manufacturers protocol. The plates were measured on a TecanSpark microplate reader at 450 nm with a wavelength correction of 540 nm. Data were fitted to 4 parameters logistic (4 PL) model and sample IL2 concentrations were calculated using GraphPad Prism 10.2.

### Quantification and statistical analysis

Statistical analysis was performed using Graphpad Prism 10.2. Significance is indicated as: ∗*p* < 0.05, ∗∗*p* < 0.01, ∗∗∗*p* < 0.001, Statistical tests used to determine significance are described in each figure legend. Data are presented as mean ± SD or ±SEM. Western blots were quantified using the Empiria Studio Version 1.3.0.83 (Licore).
