# Supplementary material for: The dual specificity phosphatase 2 act as distal regulatory node in T cell signaling
Source: iScience. 2026 Jul 15;29(8):116690. doi: 10.1016/j.isci.2026.116690 (PMC13383867; doi:10.1016/j.isci.2026.116690)
Supplement: Document S1. Figures S1–S5 and Table S1 [file mmc1.pdf]

iScience, Volume *29*

Supplemental information

The dual specificity phosphatase 2 act as distal regulatory node in T cell signaling

Henrike Bruckmueller, Anangi Balasiddaiah, Sofia Malek, Victoria Tenhaken, Julien Bruckmueller, Jakob Mejlvang, Helene Spangenberg, Farah Syed, Bjarne Johansen, Hanne Kildalsen, Ingolf Cascorbi, and Ole-Morten Seternes

Figure S1

A

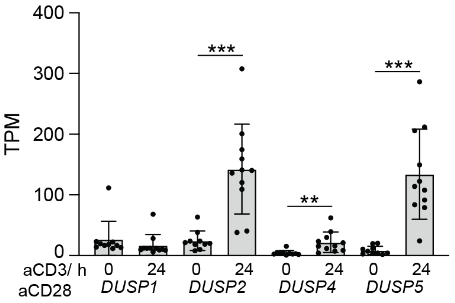

B

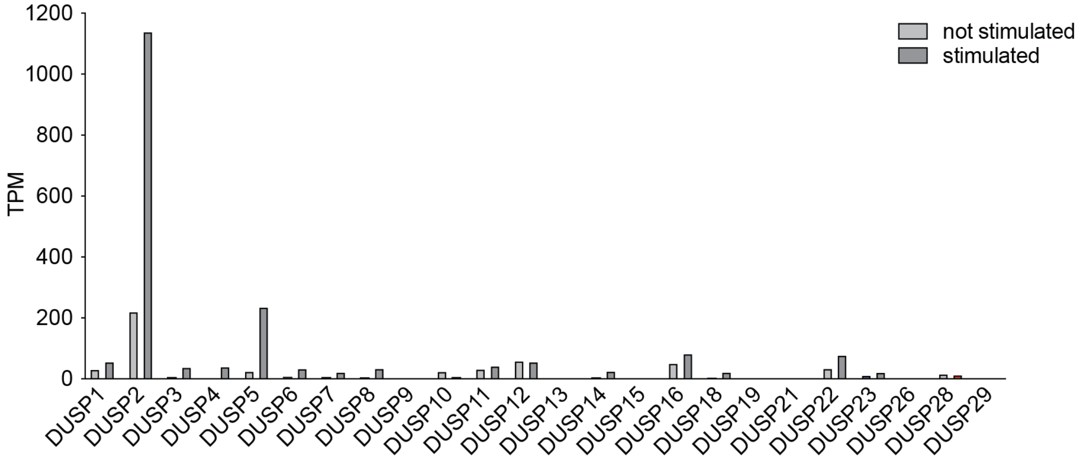

C

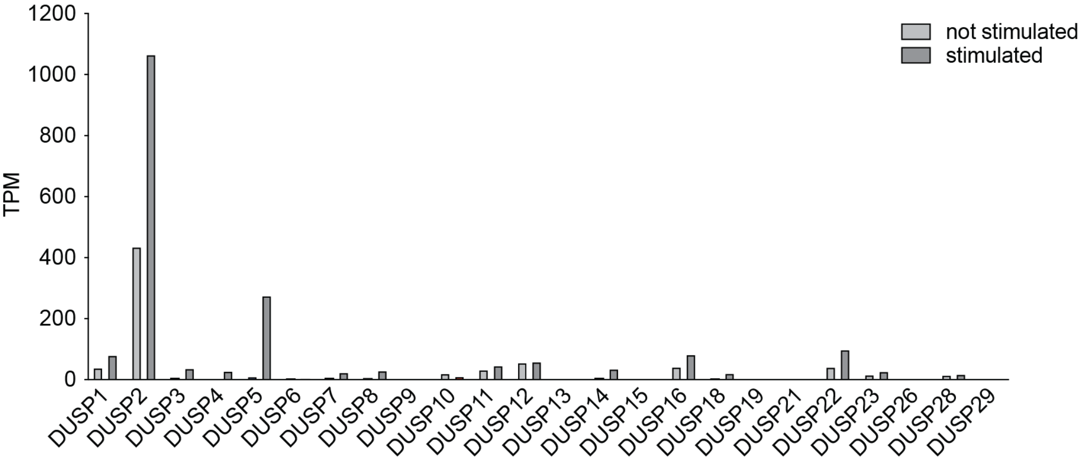

Figure S1. *DUSP2* is highly expressed in the cells of hematological origin and the highest expressed DUSP in primary human CD4$^+$ and CD8$^+$ cells.

A)Gene expression data of *DUSP1, 2, 4, 5* from primary human CD4$^+$T cells unstimulated (0h) or stimulated with aCD3/aCD28 for 24h were extracted from the GSE69549 (14) and are presented as mean TPM ± SD from three independent donors (n = 10). Expression data of all

DUSPs detected via RNAseq in primary human $CD4^+$ cells (B) and primary human $CD8^+$ cells (C) not stimulated or stimulated with aCD3/aCD28 coated beads. The data are presented as transcripts per million (TPM) and are derived from 91 healthy subject from the Schmiedel et al. data set (44). All data sets are derived from the human protein atlas (www.proteinatlas.org). Unpaired students t-test, ** $p < 0.01$, *** $p < 0.001$

Figure S2

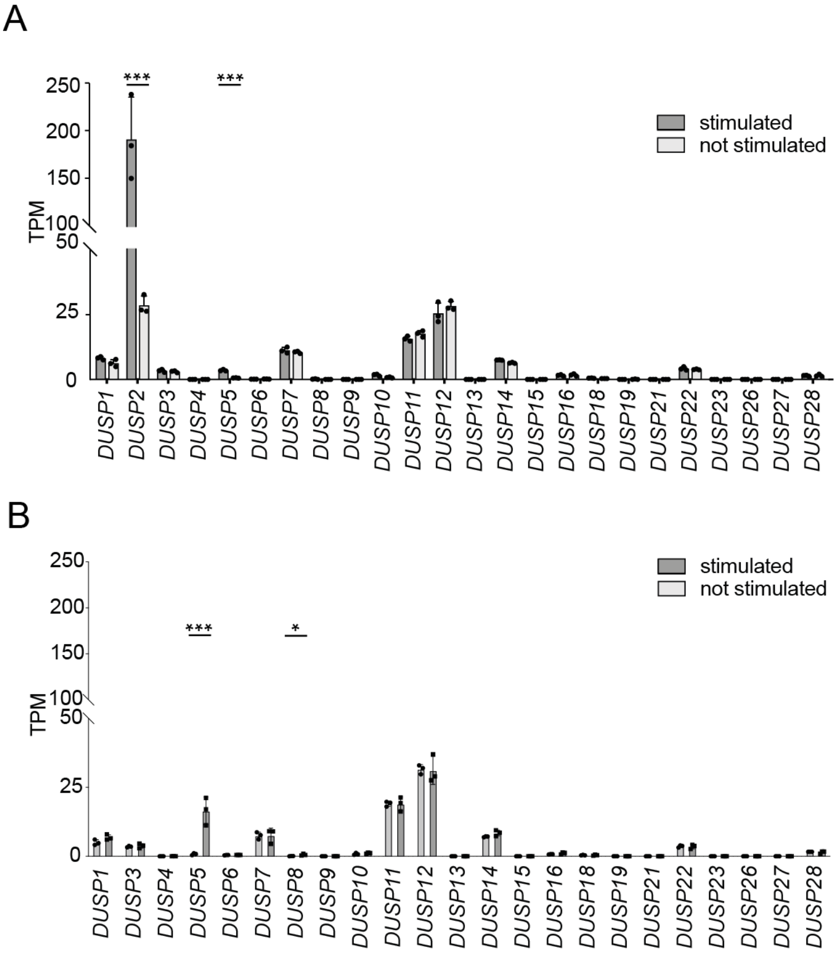

Figure S2. Expression of all detectable DUSPs in Jurkat and Jurkat DUSP2^{KO} cells

A) Expression levels (TPM) of all detectable DUSPs identified in the RNAseq analysis of Jurkat cells unstimulated or stimulated for 2h with aCD3/aCD28 coated beads (bead/cell ration 1:1) (n=3). B) Expression levels (TPM) of all detectable DUSPs identified in the RNAseq analysis of Jurkat DUSP2^{KO} cells unstimulated or stimulated for 2h with aCD3/aCD28 coated beads (bead/cell ration 1:1) (n=3). Since 0,0014% of the read of one unstimulated Jurkat sample could be aligned to potential mycoplasma reads these data need to be interpreted with care. However, the results for DUSP2 and DUSP5 were replicated with various stimuli in mycoplasma free cells. For all experiments cell were starved overnight. Expression data are presented as mean ± SD. Unpaired student t-test, * $p<0.05$, , *** $p<0.001$; TPM = Transcripts per million

Figure S3

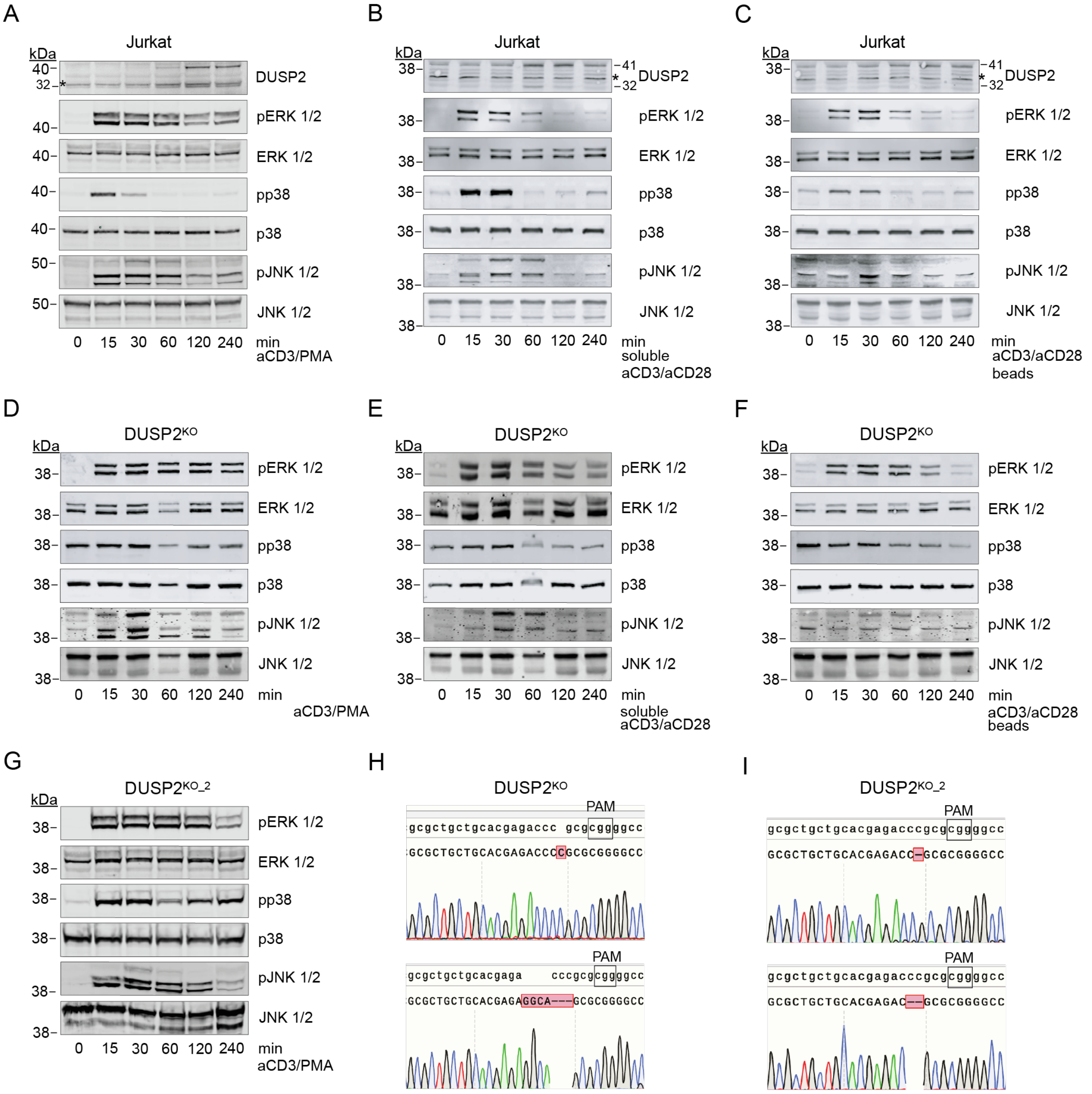

Figure S3. DUSP2 activity in Jurkat T cells on MAPKs upon different stimuli

A-E) Jurkat or DUSP2^{KO} cells were starved overnight and thereafter stimulated with either 5 µg/ml aCD3 and 10 nM PMA (A, D), 1 µg/ml soluble aCD3 and 1 µg/ml soluble aCD28 (B, E) or aCD3/aCD28 coated beads in a 1:1 bead/cell ratio (C, F) for the indicated time points. The impact of DUSP2 loss on MAPK phosphorylation was further confirmed in an additional Jurkat DUSP2 knock-out clone (G) Jurkat DUSP2^{KO_2} after overnight starving and stimulation with 5 µg/ml aCD3 and 10 nM PMA. Sanger sequencing revealed a successful genome editing by

CRISPR/Cas9 for Jurakt DUSP2^{KO} cells (H), sequence of the two individual edited alleles, shown as electrochromatograms in upper and lower panels respectively, with sequence of mutated allele in Upper case letters, and Jurkat DUSP2^{KO_2} (I) sequence of the two individual edited alleles, shown as electrochromatograms in upper and lower panels respectively, with sequence of mutated allele in Upper case letters. Western blot data were visualized using the following antibodies: a-DUSP2 (custom made), a-pERK1/2 (CST, #9101), a-ERK1/2 (Santa Cruz, sc514302), a-pp38α (CST, #9211), a-p38 (Santa Cruz, sc7972), a-pJNK1/2 (Promega, V7938), a-JNK1/2 (Santa Cruz, sc-7345). Marker = Chameleon Duo Pre-stained Protein Ladder (928-60000, Licor), PMA = phorbol-12-myristate-13-acetate.

Figure S4

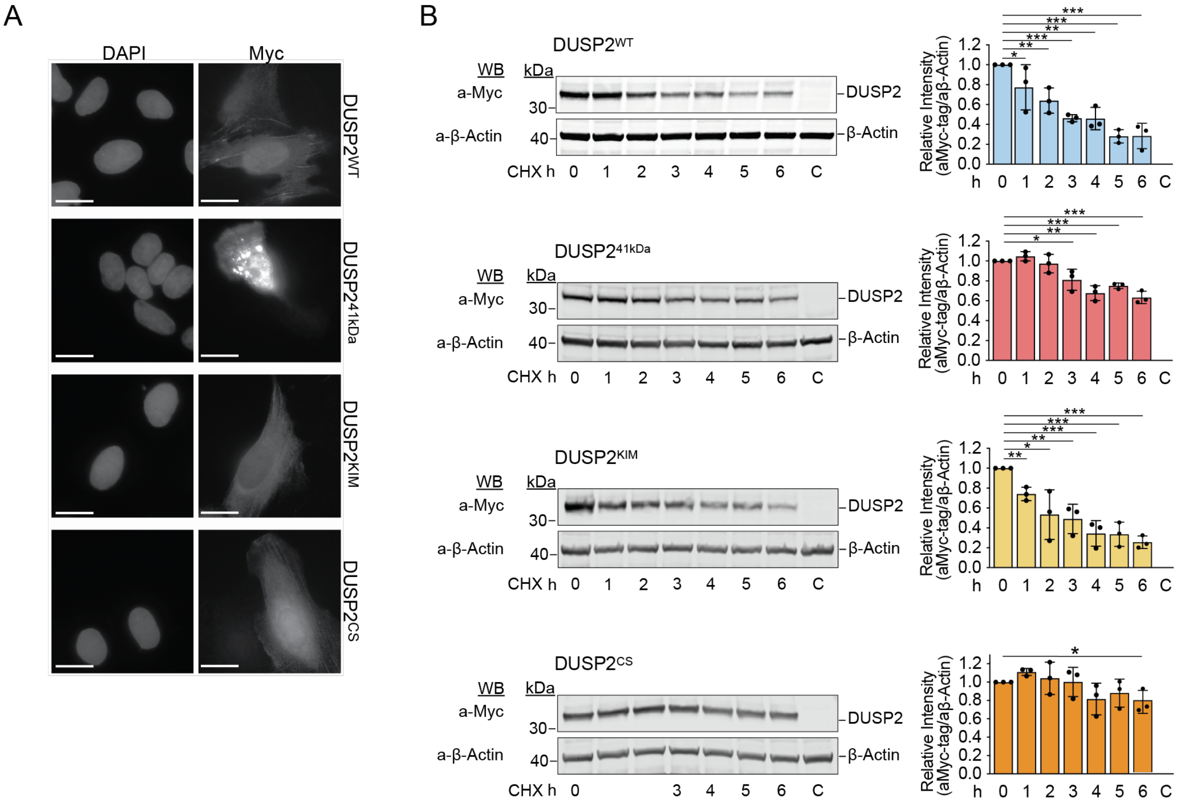

Figure S4. The extended DUSP2 protein forms aggregates and shows a higher stability compared to DUSP2^{WT} protein.

A) Microscopic analysis showed U2OS cells transfected with myc-tagged expression vectors encoding either DUSP2^{WT}, DUSP2^{CS}, DUSP2^{KIM}, or DUSP2^{41kDa} and stained with an anti-myc antibody to detect DUSP2. The scale bar indicated a length of 25 µm. B) Western blot analysis of protein stability experiments in Hek cells transfected with the different variants of Myc-tagged DUSP2 protein (DUSP2^{WT}, DUSP2^{CS}, DUSP2^{KIM}, or DUSP2^{41kDa}) before treatment with 10 µM CHX for the indicated time points. Data were visualized using the following antibodies: a-Myc-tag (Sigma-Aldrich, SAB4301136), a-β-Actin (CST, #4968). Data are presented as mean ± SD of densitometric analysis of western blots signals, n = 3, students t-test, * $p < 0.05$, ** $p < 0.01$. C, untransfected control, CHX = cycloheximide

Figure S5

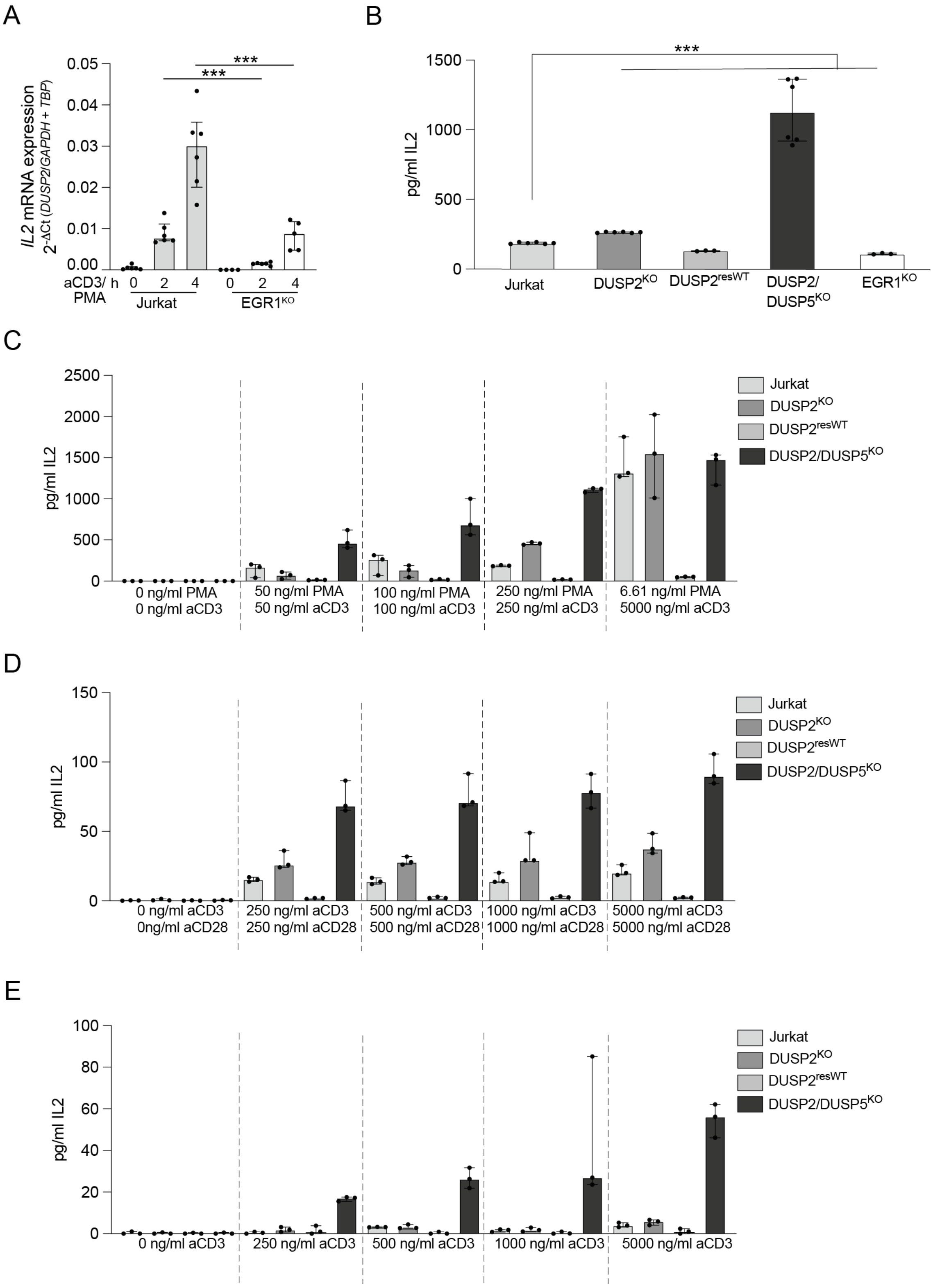

A
IL2 mRNA expression
2-ΔCt (DUSP2/GAPDH + TBP)
0.05
0.04
0.03
0.02
0.01
0.00
***
***
aCD3/ h
PMA
0
2
4
Jurkat
EGR1KO
B
pg/ml IL2
1500
1000
500
0
***
Jurkat
DUSP2KO
DUSP2resWT
DUSP2/
DUSP5KO
EGR1KO
C
pg/ml IL2
2500
2000
1500
1000
500
0
0 ng/ml PMA
0 ng/ml aCD3
50 ng/ml PMA
50 ng/ml aCD3
100 ng/ml PMA
100 ng/ml aCD3
250 ng/ml PMA
250 ng/ml aCD3
6.61 ng/ml PMA
5000 ng/ml aCD3
Jurkat
DUSP2KO
DUSP2resWT
DUSP2/DUSP5KO
D
pg/ml IL2
150
100
50
0
0 ng/ml aCD3
0ng/ml aCD28
250 ng/ml aCD3
250 ng/ml aCD28
500 ng/ml aCD3
500 ng/ml aCD28
1000 ng/ml aCD3
1000 ng/ml aCD28
5000 ng/ml aCD3
5000 ng/ml aCD28
Jurkat
DUSP2KO
DUSP2resWT
DUSP2/DUSP5KO
E
pg/ml IL2
100
80
60
40
20
0
0 ng/ml aCD3
250 ng/ml aCD3
500 ng/ml aCD3
1000 ng/ml aCD3
5000 ng/ml aCD3
Jurkat
DUSP2KO
DUSP2resWT
DUSP2/DUSP5KO

Figure S5. DUSP2 and DUSP5 act in concert in controlling IL2 production in T cells

A)Analysis of mRNA expression of *IL2* using qRT-PCR in Jurkat and DUSP2^{KO} cells stimulated with aCD3/PMA for 0-6h. Gene expression data are presented as target gene mRNA expression normalized to geometric mean of GAPDH and TBP as control. For all experiments cell were starved overnight, and all experiments were performed at least in replicates. B) ELISA analysis indicates the IL2 secretion of second clones of DUSP2^{KO}, DUSP2^{resWT} and DUSP/DUSP5^{KO} as well as two clones of DUSP2^{resKIM} and one clone of EGR1^{KO} cells 24h after stimulation with aCD3/PMA. The Jurkat cell data are the same as presented in Fig. 6D and are only included for comparison. C) ELISA analysis of IL2 secretion of Jurkat, DUSP2^{KO}, DUSP2^{resWT} and DUSP/DUSP5^{KO} cells either unstimulated or after 24 h of stimulation with four different concentrations of aCD3/PMA. D) ELISA analysis of IL2 secretion of Jurkat, DUSP2^{KO}, DUSP2^{resWT} and DUSP/DUSP5^{KO} cells either unstimulated or after 24 h of stimulation with four different concentrations of aCD3/aCD28. E) ELISA analysis of IL2 secretion of Jurkat, DUSP2^{KO}, DUSP2^{resWT} and DUSP/DUSP5^{KO} cells either unstimulated or after 24 h of stimulation with four different concentrations of aCD3. All experiments were performed at least in 3 replicates, data are presented as mean ± SD, students t-test, *** $p < 0.001$. PMA = phorbol-12-myristate-13-acetate, concentrations used: 250ng/ml aCD3 + 250ng/ml PMA

Table S1 Oligonucleotides and sequence based reagents

**Oligonucleotides and sequence-based reagents***	
**Name**	**Sequence (5’ à 3’) / identifier**
DUSP2-InF_F	GATATCTCGAGGATCCatggggctggaggcg
DUSP2-InF_R	ACTAGTGCGGCCGCGGTACCtcagtgacacagcacctggg
DUSP2_41kDa_InF_R	ACTAGTGCGGCCGCGtcaccccagcagaagagcacc
Egr1promF	TATCGAATTCCCACGACGGAGGGAATAGCCTTTCGA
Egr1promR	CGACCGGTGATCCCCCGAGAACTGATGTTGGGTGG
V5-tagF	CTAGACTATGCTGTACTGTCGAGACCTAGGAGTGGGTTT GGGATTGGCTTTCCC
V5-tagR	TCGAGGGAAAGCCAATCCCAAACCCACTCCTAGGTCTCG ACAGTACAGCATAGT
3xFLAG-tagF	TCGAGGACTACAAGGACCACGACGGCGATTATAAGGATC ACGACATCGACTACAAAGACGACGATGACAAGTAGT
3xFLAG-tagR	CTAGACTACTTGTCATCGTCGTCTTTGTAGTCGATGTCGT GATCCTTATAATCGCCGTCGTGGTCCTTGTAGTCC
gRNA DUSP2	GCTGCTGCACGAGACCCGCG
gRNA DUSP5	GAGCGAGCCGCGCACGTTCG
gRNA EGR1	GCGGCCAGTATAGGTGATGG
CpG	(DNA-ODN) ZOEZOEZZZZEZOEZZZZOEZT
TaqMan assay for DUSP2	Assay ID: Hs00358879_m1
TaqMan assay for DUSP5	Assay ID: Hs00244839_m1
TaqMan assay for EGR1	Assay ID: Hs00152928_m1
TaqMan assay for IL2	Assay ID: Hs00174114_m1
TaqMan assay for GAPDH	Assay ID: Hs00266705_g1
TaqMan assay for TBP	Assay ID: Hs00427620_m1

*All oligos and sequence based reagents were purchased from Sigma-Aldrich, Merck, Darmstadt, Germany and the TaqMan Assays are from ThermoFisher Scientific, Waltham, USA

References

14. Quinn, E.M., et al., Transcriptome Analysis of CD4+ T Cells in Coeliac Disease Reveals Imprint of BACH2 and IFNgamma Regulation. PLoS One, 2015. 10(10): p. e0140049. doi: 10.1371/journal.pone.0140049.

44. Schmiedel, B.J., et al., Impact of Genetic Polymorphisms on Human Immune Cell Gene Expression. Cell, 2018. 175(6): p. 1701–1715 e16. doi: 10.1016/j.cell.2018.10.022.
